# Climate state controls the strength of the latitudinal diversity gradient

**DOI:** 10.1098/rspb.2026.1490

**Published:** 2026-10-28

**Authors:** Die Wen, Lewis A. Jones, Junxuan Fan, Philip D. Mannion

**Affiliations:** ^1^ School of Earth Sciences and Engineering and Frontiers Science Center for Critical Earth Material Cycling, Nanjing University, Nanjing 210023, People's Republic of China; ^2^ State Key Laboratory of Critical Earth Material Cycling and Mineral Deposits, Nanjing University, Nanjing 210023, People's Republic of China; ^3^ Department of Earth Sciences, University College London, London WC1E 6BT, UK

**Keywords:** deep time, macroevolution, biodiversity, latitudinal diversity gradient, climate states, Phanerozoic, Palaeozoic, Mesozoic, Cenozoic

## Abstract

The latitudinal diversity gradient (LDG)—a decline in taxonomic richness from the Equator to the poles—is a fundamental biogeographic pattern on Earth today, yet its primary regulators remain debated. Whereas many of the proposed climatic and environmental drivers covary with latitude in the present day, these variables have fluctuated substantially over millions of years. The fossil record therefore offers a unique perspective on the response of diversity and, by extension, the LDG, to global climatic change. We test the hypothesis that Earth's long-term climate state controls LDG strength, determining the conditions under which steep, ‘modern-type’ gradients with low-latitude-centred diversity peaks emerge. By analysing sampling-standardized marine invertebrate diversity across the past 500 million years, we reveal a systematic link: ‘modern-type’ LDGs typically prevail during coldhouse–coolhouse intervals, whereas flattened LDGs or temperate diversity peaks occur predominantly within transitional, warmhouse and hothouse climates. This robust phenomenon demonstrates that long-term climate exerts a primary control on global biodiversity patterns, whereas palaeogeography, including the distribution of shallow-marine habitat and continental configuration, modulates the regional expression of the LDG, including hemispheric variation. As anthropogenic warming pushes Earth towards hothouse conditions, our results offer a critical geohistorical perspective on the impending restructuring of global biodiversity.

## 1. Introduction

Life on Earth is most diverse at low latitudes, with the number of species found in a given area decreasing towards the poles. This latitudinal diversity gradient (LDG) is one of Macroecology's longest recognized patterns [1–3]. Although there is evidence for weak bimodality, including shallow tropical diversity depressions [4], the otherwise steep LDG, with a low-latitude-centred peak, is remarkably prevalent and has been observed across most taxonomic groups in both terrestrial [3,5] and marine [6] realms. Understanding what regulates this gradient is essential for predicting how the distribution of diversity might respond to the ongoing climate emergency. However, after two centuries of study, the answer remains elusive.

Part of the problem is that many of the proposed climatic and environmental drivers of the LDG covary with latitude in the present day. By contrast, these variables have fluctuated substantially over long periods in deep time. Consequently, the fossil record offers a unique perspective by providing long-term empirical evidence on the response of diversity and, by extension, the LDG, to global climatic change [7–11]. Sampling-standardized estimates of diversity suggest that the steep ‘modern-type’ gradient, with a low-latitude-centred peak observed today, was not always present, with flattened and even strongly bimodal gradients observed across a range of taxonomic groups at various points in Earth's history [7,12–15]. One proposed explanation for this temporal variation in the strength and shape of the LDG is concurrent shifts in global climate state [14].

Recent work has categorized the Phanerozoic climate states into five types: (i) coldhouse, with a strong latitudinal temperature gradient and extensive polar ice; (ii) coolhouse, with a moderate temperature gradient and reduced ice cover; (iii) transitional, representing intermediate conditions; (iv) warmhouse, with a flattened temperature gradient and ice-free poles; and (v) hothouse, characterized by a uniformly weak latitudinal temperature gradient and no polar ice [16]. A critical synthesis of the literature has suggested that ‘modern-type’ LDGs might be limited to coldhouse–coolhouse climate states, with the tropical concentration of diversity primarily dictated by a strong Equator-to-pole temperature gradient and the presence of polar ice caps [14,17,18]. Although recent work has examined Phanerozoic variation in LDG strength [7], finding only weak support for shallower gradients during warmer intervals, it remains unclear whether LDG strength varies systematically across more finely resolved climate states and whether this relationship is consistently expressed in both hemispheres.

Here, we test the hypothesis that Earth's long-term climate state governs the strength of LDGs through time, with ‘modern-type’ LDGs occurring primarily during coldhouse–coolhouse intervals. We estimate hemisphere-specific LDG slopes for each geological stage across the Phanerozoic using global fossil occurrence data of marine invertebrates and sampling-standardization approaches. By integrating with independently defined global climate states, we assess whether the strength of the LDG varies systematically with climate state and evaluate the role of long-term temperature fluctuations in structuring the distribution of diversity. This hemisphere-specific approach also allows us to determine whether LDGs were globally coherent or hemispherically decoupled and to identify palaeogeographic deviations from the expected climate-state pattern.

## 2. Data and methods

### (a) Fossil occurrence data

Genus-level fossil animal occurrence data spanning the Tremadocian to the Holocene were downloaded from the Paleobiology Database (PBDB) on 3 August 2026 using the following URL: https://paleobiodb.org/data1.2/occs/list.csv?base_name=Animalia%5EVertebrata&taxon_reso=genus&idtype=latest&taxon_status=valid&idqual=genus_certain&pres=regular&interval=Tremadocian%2CHolocene&envtype=marine&show=genus%2Cpres%2Cstrat%2Ccoll%2Ccoords%2Cloc%2Cclass%2Cenv%2Cresgroup. The query excluded vertebrates and returned 860 604 genus-level occurrence records. The downloaded data were subsequently restricted to eight major and representative marine invertebrate groups: Mollusca, Arthropoda, Brachiopoda, Bryozoa, Echinodermata, Cnidaria, Porifera and Graptolithina. These data were retrieved using the PBDB API service and were restricted to marine environments, valid taxa and regular preservation (i.e. excluding form taxa and ichnotaxa), based on the latest taxonomic opinions at genus level. The raw data were preserved in their original state and archived alongside the processed dataset, along with the acquisition and processing code to ensure full reproducibility [19] (see the ‘Data accessibility’ statement).

All fossil occurrences were assigned to stage-level time bins following the International Chronostratigraphic Chart v. 2024/12 (https://stratigraphy.org/chart), except for the Pleistocene and Holocene, which were evaluated at the epoch level owing to the prevalence of fossil occurrence data not resolved to stage level for these intervals (supplementary material, table S2). Temporal binning was implemented using the ‘majority’ approach via the bin_time() function from the palaeoverse R package v. 1.3.0 [20]. This approach uses the age range of occurrences to assign them to the time bin with which they temporally most overlap. All occurrences with less than 50% of their age range within their time bin were discarded. To minimize differences in spatio-temporal resolution, such as the lumping or splitting of geological sections into PBDB collections, we also rounded geographic coordinates to two decimal places and combined collections from the same time and place, removing duplicated occurrences arising as a result. Finally, genus names were standardized by removing any suffixes (e.g. subgenus) to prevent the counting of non-unique genera.

To enable palaeogeographic analyses, palaeo-coordinates were estimated for each fossil collection using the PALEOMAP model [21] via the palaeorotate() function in the palaeoverse package v. 1.3.0 [20]. Reconstructions were based on the present-day longitude and latitude of each collection and the midpoint age of the assigned time bin. Any collections for which palaeo-coordinates could not be estimated were excluded from the dataset. Subsequently, using the estimated palaeo-coordinates, we assigned fossil collections to cells within a global equal-area hexagonal grid (250 km spacing between cell centroids) generated via the dggridR package v. 3.1.1 [22]. Following data filtering and processing, the final dataset contains 23 211 genera across 575 419 occurrences from 110 684 collections.

### (b) Reconstructing the latitudinal diversity gradient

To reconstruct the LDG, while accounting for differences in spatio-temporal sampling, we estimated genus richness for each grid cell within each time bin using coverage-based interpolation and extrapolation of genus richness. For each cell–time-bin combination, the input vector consisted of the total number of collections followed by the incidence frequency of each genus, defined as the number of collections in which that genus occurred. To implement this, we used the estimateD() function from the iNEXT R package v. 3.0.1 [23], specifying incidence-frequency data (datatype = ′incidence_freq′), a target coverage of 0.7 and a Hill-number order of *q* = 0. The complete code used to generate these estimates, together with the resulting genus-richness data, is available in the Zenodo repository ( https://doi.org/10.5281/zenodo.17631059). Prior to generating estimates, we excluded cells with fewer than five fossil occurrences as a data quality control (QC) step to ensure sufficient samples for robust estimates. After generating the estimates, we discarded any extrapolated values with an estimated sample size of more than double the observed sample size in order to retain only the robust estimates [24].

We normalized diversity estimates within each time bin by dividing each cell's richness by the maximum richness observed in that time bin and then multiplying by 100, before binning cells into 12 equal-area palaeolatitudinal bins. To estimate the strength of the LDG, we used a per-cell resampling regression approach. For each time bin and hemisphere, we randomly sampled one grid cell from each occupied palaeolatitudinal bin and performed ordinary least squares regression of the normalized richness values of the sampled cells against the absolute midpoint of the corresponding palaeolatitudinal bins. This procedure was repeated 100 times for each time bin and hemisphere, generating a distribution of LDG slopes. We used the median of these resampled slopes as the final LDG slope estimate and used the central 95% interval of resampled slopes to evaluate uncertainty (supplementary material, figures S4–S7).

Prior to analysis, to ensure reliable slope estimates, we discarded any hemispheres without adjacent palaeolatitudinal bins within the tropical (0–30°) and temperate (30–60°) bands, as well as those with fewer than three palaeolatitudinal bins in total (supplementary material, figure S2).

We used the median of the 100 resampled slopes as the final LDG slope estimate. Based on the median value, slopes below −0.1 were classified as negative, slopes between −0.1 and 0.1 (inclusive) as near-zero and slopes above 0.1 as positive. These categories describe the direction and magnitude of the median slope estimate.

For each stage and hemisphere, the median of the 100 resampled slope estimates was used as the representative LDG slope, and the 2.5th and 97.5th percentiles defined the central 95% resampling interval. The width of this interval summarizes the variability of slope estimates arising from spatial resampling.

### (c) Quantifying the relationship between climate state and latitudinal diversity gradients

To examine the relationship between LDG slope and palaeoclimatic conditions, we assigned slope estimates to climate states based on their respective stratigraphic bin midpoints. We followed the stage-level climate-state designations proposed by Judd *et al.* [16], which include coldhouse, coolhouse, transitional, warmhouse and hothouse conditions. We then calculated the median LDG slope for each climate state in each hemisphere to provide an overview of the general diversity trend across palaeoclimatic conditions. To further evaluate differences in LDG slopes across these climate states, we conducted pairwise Wilcoxon rank-sum one-sided tests using the wilcox.test() function available in base R. These tests were performed both with slopes from the two hemispheres pooled and separately for the Northern and Southern Hemispheres. These tests assessed whether LDG slope estimates are numerically lesser in cooler climate states than in warmer ones. To account for multiple comparisons and control the false discovery rate, we adjusted the *p*-values using the Benjamini–Hochberg (BH) procedure, thereby reducing the risk of false positives and ensuring the reliability of the results.

## 3. Results

Overall, after palaeogeographic reconstruction, fossil occurrences show a greater representation in the Southern Hemisphere during the Palaeozoic, whereas the Northern Hemisphere is better represented from the Mesozoic onwards (figure 1; supplementary material, figure S1). Using strict data QC steps (see §2 and supplementary methods), slope estimates can be generated for at least one hemisphere in 83 out of 87 of our Phanerozoic stage-level time bins. Of these, 70 and 72 time bins have sufficient data (i.e. having adjacent fossil-bearing palaeolatitudinal bins in both tropical and temperate regions and at least three palaeolatitudinal bins in total) to generate slope estimates for the Northern and Southern Hemispheres, respectively. These estimates are fairly evenly distributed across climate states, with 24 in coldhouse, 30 in coolhouse, 33 in transitional, 28 in warmhouse and 27 in hothouse intervals (figure 2). Importantly, most well-sampled stage–hemisphere spatio-temporal bins extend to at least 60°, and many reach at least 70°, indicating broad high-latitude coverage for estimating LDG slopes (supplementary material, figure S3). Sensitivity analyses confirm that the LDG slope results are broadly robust. Different grid resolutions, palaeolatitudinal binning schemes, occurrence- or collection-based filters and resampling versus direct all-cell estimation all produce highly consistent slope estimates (supplementary material, figures S10 and S11). The 95% resampling intervals vary in width, generally widening in the Palaeozoic time bins relative to those of the Mesozoic. This indicates that slope estimates are more sensitive to spatial resampling during many Palaeozoic intervals (figure 1).

**Figure 1.**
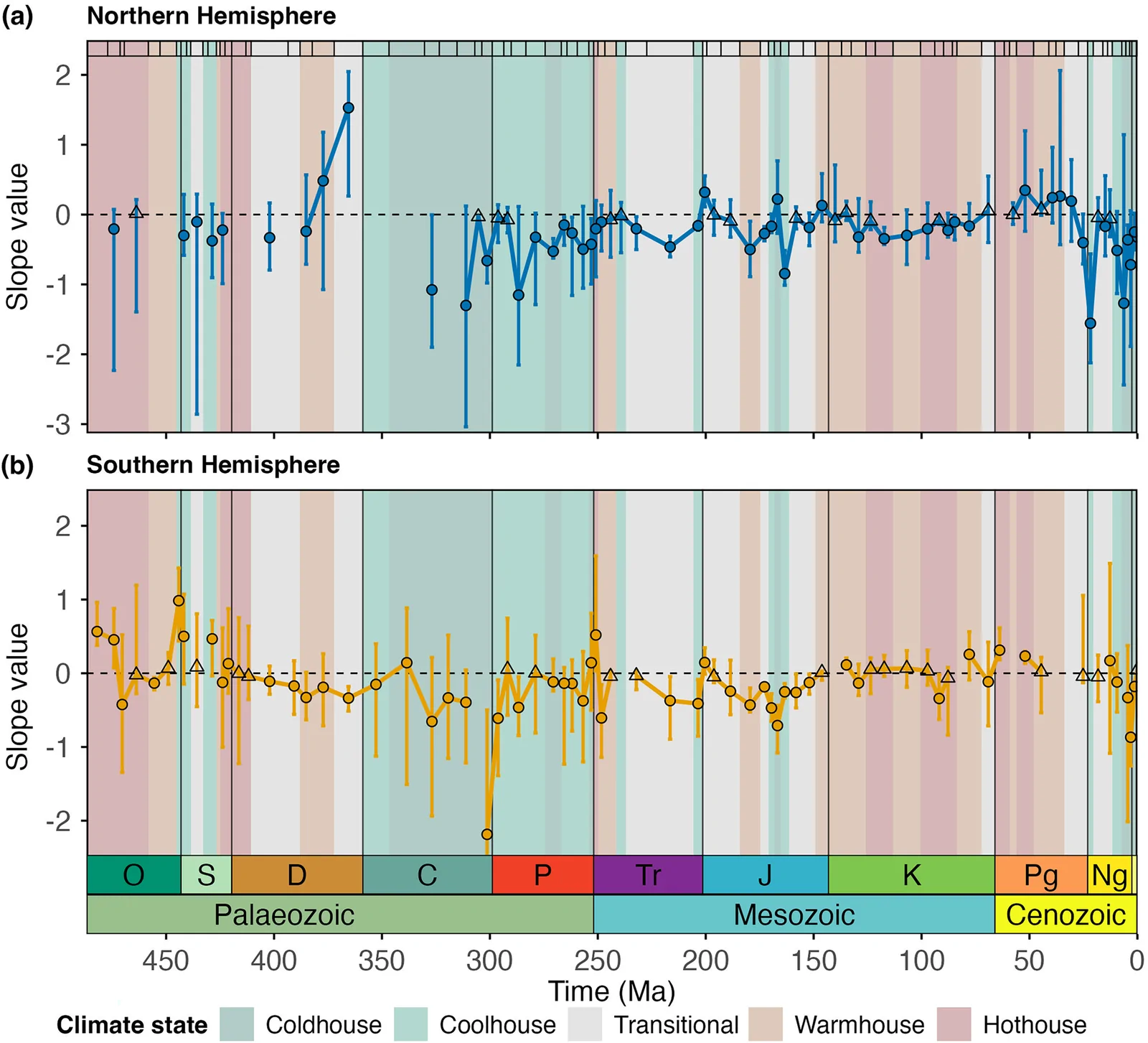
Phanerozoic LDG slopes for the Northern (a) and Southern (b) Hemispheres. LDG slope reflects changes in reconstructed sampling-standardized genus richness from the Equator to the poles, based on richness values normalized to a 0–100 scale. Normalization was performed within each stage by dividing the richness of each cell by the maximum richness across all cells in that stage, then multiplying by 100. Normalized cell values were then aggregated into 12 equal-area latitudinal bands. For each stage, LDG slopes were estimated through repeated resampling. Points and lines show the median resampled slope, and error bars show the central 95% resampling interval, defined by the 2.5th and 97.5th percentiles of the resampled slope distribution. For visualization and descriptive interpretation, LDG slope median values between −0.1 and 0.1 were defined as ‘near-zero’ and are shown as triangles. Slopes below −0.1 are interpreted as hemisphere-specific modern-type LDG, in which richness decreases from low to high absolute palaeolatitudes. This definition does not require a strictly unimodal equatorial peak and allows for weak bimodality or shallow tropical diversity depressions. Slopes greater than or equal to −0.1 were classified as non-modern-type LDGs, encompassing near-zero patterns with little overall directional change in richness, patterns in which richness peaks at temperate latitudes and declines towards both lower and higher latitudes or positive gradients in which richness even increases towards higher latitudes. Climate states [16] are shown as coloured bars: coldhouse (dark green), coolhouse (light green), transitional (grey), warmhouse (light brown) and hothouse (dark brown). Geological system: O, Ordovician; S, Silurian; D, Devonian; C, Carboniferous; P, Permian; Tr, Triassic; J, Jurassic; K, Cretaceous; Pg, Palaeogene; Ng, Neogene; Q, Quaternary.

**Figure 2.**
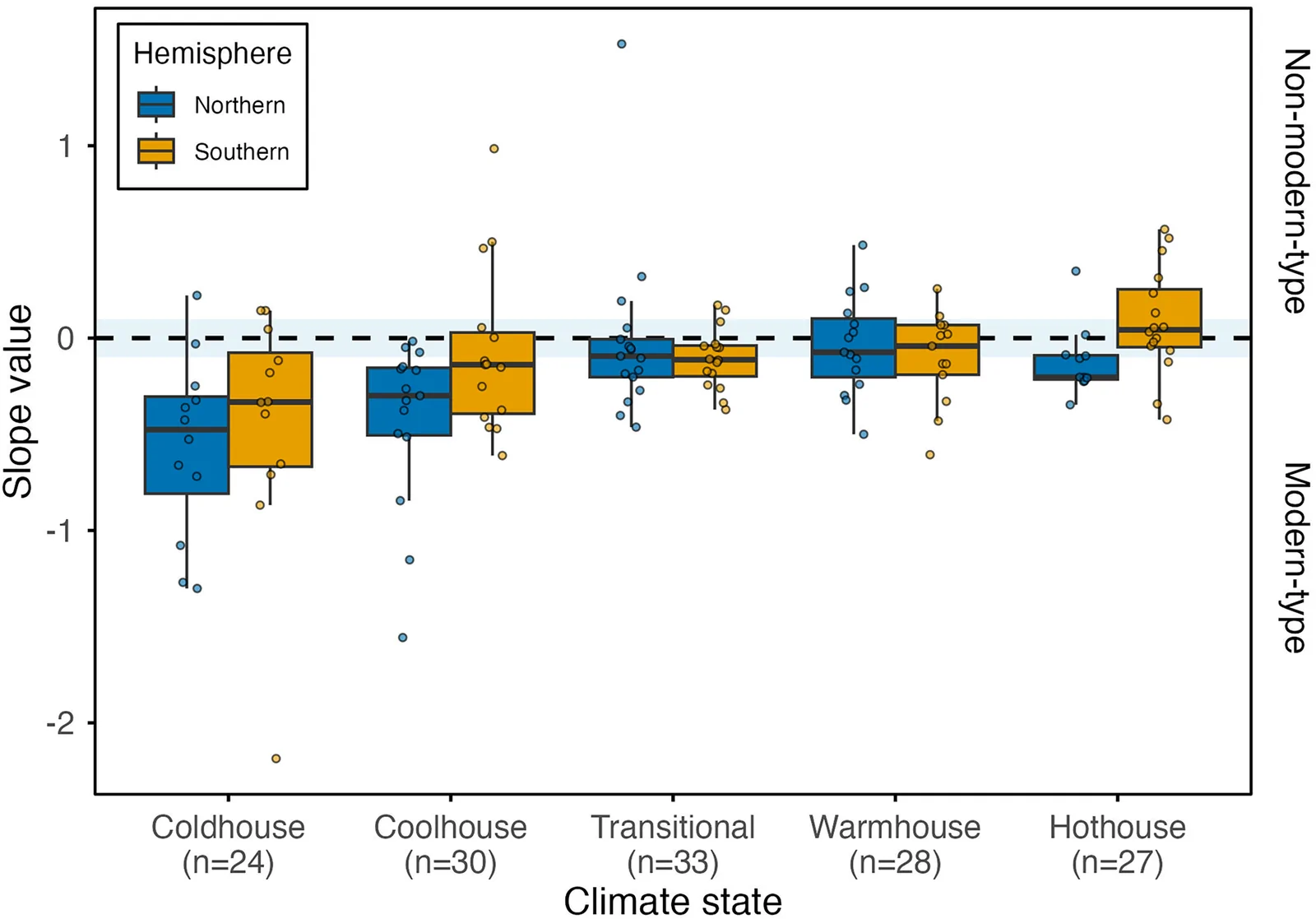
Estimated LDG slopes grouped by climate state [16]. Box plots show LDG slopes under different climate states for the Northern and Southern Hemispheres. Coloured points indicate the LDG slope for each geological stage. The blue-shaded area highlights slope estimates of small magnitude, operationally defined here as ‘near-zero’ slopes with values between −0.1 and 0.1, indicating little overall directional change in richness across palaeolatitudes. Accordingly, all slopes greater than or equal to −0.1 were classified as non-modern-type LDGs, whereas those below −0.1 were classified as modern-type LDGs.

Although ‘modern-type’ LDG patterns are common, they are not universally expressed across all intervals of the past 500 million years. Instead, LDG slopes exhibit substantial variation, including intervals with a steep, negative slope, consistent with a hemisphere-specific ‘modern-type’ gradient, as well as intervals with a ‘non-modern-type’ gradient, ranging from a near-zero slope, indicating a flattened gradient with little variation in richness across latitudes, to a strongly positive slope that reflects peak richness away from the Equator (figure 1).

The Northern and Southern Hemisphere slopes are not always consistent. During some intervals, the two hemispheres exhibit opposite slope directions or markedly different slope strengths, indicating that the LDG can be hemispherically decoupled (supplementary material, figure S9a). We quantified sampling evenness using three metrics: sampling-profile dissimilarity (how differently sampled cells are distributed among latitudinal bins between hemispheres), hemispheric differences in the standard deviation of *k* across latitudinal bins and differences in median *k* between hemispheres, where *k* denotes the number of sampled cells within each latitudinal bin. Sampling-profile dissimilarity is not significantly correlated with either the absolute difference in slope between hemispheres or directional hemispheric slope difference (supplementary material, figures S8 and S9b and table S3). The absolute slope difference is significantly negatively correlated with median *k* asymmetry (ρ = −0.336, *p*-value = 0.009; supplementary material, table S3).

LDG slopes in the Palaeozoic are characterized by temporal variability, relatively wide 95% resampling intervals and, especially, hemispheric differences (figure 1). In the Ordovician, the Southern Hemisphere shows a range of slopes, varying from shallow positive and near-zero (−0.1 to 0.1), to shallow negative, contrasting with predominantly negative slopes in the Northern Hemisphere. This hemispheric contrast continues into the Silurian, when Southern Hemisphere slopes fluctuate but remain predominantly positive or near-zero, whereas slopes remain negative in the Northern Hemisphere. Low sampling in the Northern Hemisphere limits reconstruction of slopes for much of the Devonian, with negative slopes in the Early and Middle Devonian and steep positive slopes in the Late Devonian. In the Early Devonian, the Southern Hemisphere slopes are generally positive but shift to shallow negative and near-zero values in the Middle and Late Devonian. The Carboniferous predominantly exhibits negative slopes across both hemispheres, including a steep negative Southern Hemisphere slope during the Late Carboniferous, although with some near-zero to shallow positive values. In the earliest and latest Permian, Northern Hemisphere slopes are near-zero, whereas they are negative in the intervening interval, with fluctuations in slope steepness. By contrast, Southern Hemisphere slopes are strongly negative in the earliest Permian but then become close to zero and shallow negative throughout most of the Permian, except for the Changhsingian (latest Permian), which exhibits a positive slope.

The Mesozoic is marked by relatively limited variation in LDG slope, narrow 95% resampling intervals and small hemispheric differences (figure 1). In the Early and Middle Triassic, there is a shift from positive to negative LDG slopes in the Southern Hemisphere, while the Northern Hemisphere remains characterized by near-zero to shallow negative slopes. In the Late Triassic through to the Early Jurassic, Southern Hemisphere LDG slopes shift from positive to negative, whereas Northern Hemisphere slopes remain near-zero to shallow negative. From the Late Triassic to the Early Jurassic, slopes are generally negative in both hemispheres, apart from a shallow positive slope in the earliest Jurassic. Slopes increase from the Middle Jurassic onwards but remain near-zero or shallow negative through the Late Jurassic, except for one shallow positive Northern Hemisphere estimate. Throughout the Cretaceous, LDG slopes are generally flat and stable. Northern Hemisphere slopes fluctuate during the earliest Cretaceous but remain negative or near-zero thereafter through the remainder of the Cretaceous. Southern Hemisphere slopes are predominantly near-zero, apart from a pronounced negative slope in the mid-Cretaceous and a positive slope in the Late Cretaceous.

Overall, the Cenozoic LDG is characterized by a switch from predominantly positive slopes during the Palaeogene to negative slopes during the Neogene, accompanied by relatively wide 95% resampling intervals (figure 1). In the early Palaeogene (Palaeocene), slopes are positive in both hemispheres. By the middle Palaeogene (Eocene), Southern Hemisphere data are limited, but the available estimates indicate shallow positive slopes in both hemispheres. During the late Palaeogene (Oligocene), the Southern Hemisphere has only one near-zero estimate, whereas Northern Hemisphere slopes shift from positive to steeply negative. During the Neogene, LDG slopes fluctuate but are predominantly negative, except for one positive Southern Hemisphere slope in the middle Neogene and one near-zero slope in the recent.

Both hemisphere-specific ‘modern-type’ slopes, defined as steep negative low-to-high latitude gradients, and ‘non-modern-type’ slopes, defined as near-zero or positive gradients, are observed throughout the Phanerozoic, with their frequency and strength varying across different climate states (figure 2). In the Southern Hemisphere, LDG slopes during coldhouse and coolhouse climates are predominantly negative, reflecting a modern-type LDG. Conversely, during warmhouse and hothouse climates, LDG slopes in the Southern Hemisphere are typically closer to zero or positive, suggesting a non-modern-type LDG. In the Northern Hemisphere, although LDG slopes are generally negative across all climate states, positive slopes occasionally occur during transitional, warmhouse and hothouse climates, particularly in the Palaeogene, suggesting a non-modern-type LDG during these intervals.

Pairwise Wilcoxon rank-sum one-sided tests (supplementary material, table S1) reveal that LDG slope values are significantly lesser (numerically) during coldhouse and coolhouse climates than transitional, warmhouse and hothouse climates, supporting a stronger tendency towards modern-type LDG slopes under cooler climates. However, no significant difference is observed between coldhouse and coolhouse states or among the transitional, warmhouse and hothouse climates. When the hemispheres are analysed separately, the Northern Hemisphere broadly reproduces the pooled pattern, with several differences between cooler and warmer climate states remaining significant after correction. By contrast, climate-state LDG differences are weaker in the Southern Hemisphere; although unadjusted *p*-values generally support more negative LDG slopes under cooler climates, only the coldhouse and hothouse comparison remains significant after correction (supplementary material, table S1). Although stricter QC criteria reduce the number of retained time-bin–hemisphere slopes (supplementary material, figure S12 and table S4), especially when requiring at least three cells per latitudinal band, the main climate-related patterns and Wilcoxon test results remain consistent. Thus, the overall pattern of more negative slopes under cooler climates and near-zero or positive slopes under warmer climates is unlikely to be driven by any single filtering or slope-estimation method (supplementary material, table S5).

Although LDG slopes generally align with global climate states, there are several notable deviations. For example, a positive slope is observed in the Southern Hemisphere during the Changhsingian (latest Permian), a coldhouse interval. Several hothouse stages—including the Floian (Early Ordovician), Dapingian (Middle Ordovician), Aptian (Early Cretaceous) and Santonian (Late Cretaceous) in the Northern Hemisphere, as well as the Barremian (Early Cretaceous) in the Southern Hemisphere—yield negative LDG slopes (supplementary material, figures S4 and S5).

## 4. Discussion

The relatively even sampling across climate states allows robust comparisons of LDG patterns under contrasting global climates. The modern-type LDG slopes observed during most intervals of the Late Palaeozoic Ice Age (LPIA, latest Devonian–early Late Permian) [25] and the Quaternary glaciation support the hypothesis that climate regulates LDG slope (figure 1) [13,15,26,27]. Our results also suggest that the present-day ‘modern-type’ LDG only became well established in the Middle Miocene [12,28], when the Earth entered a consistent coldhouse climate state [16]. The shift from shallow positive slopes to steep negative slopes during the Palaeogene–Neogene transition highlights the role of long-term global cooling in establishing a ‘modern-type’ LDG [18]. Our finding that Phanerozoic LDGs were generally shallower during warmer intervals is consistent with the results of a recent study [7]. However, that study combined both hemispheres and aggregated regional diversity estimates by broad climate category before estimating LDG slopes. By contrast, we estimated stage-level slopes separately for each hemisphere before comparing them across five climate states, thereby resolving temporal and hemispheric variation in the climate–LDG relationship. Both hemispheres tend to show more negative LDG slopes under cooler climates, but this climate-state signal is more consistently expressed in the Northern Hemisphere. After multiple-testing correction, only the coldhouse–hothouse contrast remains significant in the Southern Hemisphere (supplementary material, table S1). Moreover, stage-level patterns sometimes exhibit opposite slopes between hemispheres, indicating hemispheric decoupling that cannot be explained by the observed differences in sampling structure (supplementary material, figures S8 and S9, table S3). Our analysis therefore illuminates how the climate–LDG relationship varies among individual stages and between hemispheres, while also emphasizing the uncertainty introduced by spatial sampling and palaeogeographic heterogeneity.

Physiological data show that most extant marine invertebrates function optimally within temperatures of approximately 5–25°C, with peak diversity and productivity near 20°C—the temperature at which metabolic efficiency, enzyme stability and global marine species richness are consistently maximized [29,30]. Under coldhouse climate states, strong Equator-to-pole temperature gradients generated cold high-latitude surface conditions and near-freezing polar marine environments [31,32]. Together with expanded sea ice and strong seasonality, these conditions would have restricted habitable shallow-marine environments and suppressed high-latitude diversity, thereby steepening the overall low-to-high latitude richness decline. This suggests that coldhouse climates steepen diversity gradients and concentrate global diversity into narrower tropical zones, while increasing environmental instability and physiological stress at high latitudes [27,33–35]. However, physiological limits derived from extant organisms may not necessarily apply directly to extinct taxa, which may have been better adapted to warmer climates. We therefore use modern physiological data as a qualitative framework rather than as fixed thermal thresholds.

In contrast with coldhouse intervals, warmhouse and hothouse intervals—such as the Ordovician in the Southern Hemisphere, the late Silurian (Ludlow and Pridoli), several Devonian intervals, the earliest Triassic, the latest Cretaceous and the Palaeogene—often exhibit non-modern-type LDG slopes. These include near-zero or positive slopes that indicate a weakened or reversed low-to-high latitude richness decline and, in some cases, suggest that richness maxima were not consistently concentrated at the lowest latitudes (supplementary material, figure S5). This pattern probably reflects shallower Equator-to-pole temperature gradients and the poleward expansion of (sub-)tropical optimal thermal conditions that fall within the approximately 5–25°C optimal ecophysiological window of organisms, which enhanced habitat suitability at mid-latitudes (e.g. for coral reefs) [36]. Meanwhile, equatorial sea-surface temperatures during Phanerozoic warmhouse and hothouse intervals averaged approximately 28–35°C [16,32], which might have exceeded thermal optima or upper tolerances for many marine invertebrates [37,38], thereby suppressing equatorial richness and preventing biodiversity maxima from being centred at the Equator [39,40]. Under such conditions, the most suitable high-diversity environments may instead have shifted towards the tropical margins or mid-latitudes, where thermal stress is lower and shallow-marine habitats are more available [35,41]. This interpretation is consistent with evidence from Cenozoic tropical marine biodiversity hotspots, which were not fixed at the Equator but shifted through time, in association with changing thermal stress, habitat availability and tectonic configuration [35,41]. Climate simulations indicate that tropical sea-surface temperatures surpassed 40°C during several hothouse intervals (e.g. late Silurian and early Eocene) [16,32], coinciding with times of low equatorial richness and near-zero to positive LDG slopes (figures 1 and 2).

Collectively, these results support the hypothesis that global climate states exert a first-order control on LDG expression, influencing its strength and shape [12–14,17,33], although this signal can be modulated or obscured by palaeogeography, sampling and methodological choices. Sensitivity analyses further indicate that the climate–LDG relationship is not dependent on a single methodological choice (supplementary material, figures S10–S12, table S5). However, several deviations from this overall climate state–LDG pattern are notable (figure 1). Some of these departures can be attributed to data limitations and sampling failure. Tropical under-sampling is a key potential bias because limited low-latitude coverage can flatten estimated slopes or even produce positive slopes, as highlighted by recent work and by our own sampling coverage summary [42] (supplementary material, figure S2). For example, the Changhsingian within the Late Permian coldhouse presents a positive gradient in the Southern Hemisphere and a negative one in the Northern Hemisphere (supplementary material, figures S4, S6 and S7). In the Holocene, a shallow positive slope in the Southern Hemisphere appears inconsistent with the well-established present-day LDG. These asymmetries probably arise from sparse low-latitude sampling, especially in the southern low latitudes, where there is almost no fossil record [43].

Other deviations are more plausibly linked to fluctuations in palaeogeography, potentially reflecting ecological and evolutionary signals shaped by large-scale tectonic and palaeogeographic processes. These deviations do not necessarily contradict climate as a primary control of LDG strength. Rather, they suggest that climate-driven diversity gradients were filtered through the spatial distribution of shallow-marine habitats and the fossil record. Climate state may set the broad thermal template for LDGs, but palaeogeography determines where suitable shelf habitats occur, where biodiversity hotspots can develop and where fossiliferous sediments are preserved and sampled. Changes in shelf area, coastline complexity and archipelagic settings can promote habitat heterogeneity and dispersal opportunities, generating biodiversity hotspots away from the Equator. This is consistent with Cenozoic ‘hopping hotspot’ models, in which marine biodiversity centres shifted geographically through time in response to tectonic reorganization, shelf-habitat expansion, coastline complexity and archipelago formation [35,41,44]. More broadly, a recent Phanerozoic-scale analysis showed that latitudinal taxonomic centres migrated northwards, in parallel with the redistribution of continental shelf area, supporting a long-term role for plate-driven habitat reorganization in shaping biodiversity patterns [45]. These palaeogeographic effects influence both biological diversity and its fossil record [46], with shelf distribution affecting habitat availability through species–area relationships, while also controlling where fossiliferous sediments are deposited, preserved and sampled.

Hemispheric decoupling shows that deep-time LDGs were not always globally symmetrical. This pattern is unlikely to be driven simply by hemispheric sampling differences, because sampling-profile dissimilarity and latitudinal-bin sampling unevenness are not significantly correlated with Northern–Southern Hemisphere slope differences, whereas the absolute difference in median cell number was significantly negatively correlated with the absolute slope difference (ρ = −0.336, p = 0.009; supplementary material, figure S9, table S3); this is contrary to the expected slope direction if greater sampling asymmetry increased hemispheric slope differences.

Continental positions, shelf area and sampling windows were unevenly distributed between hemispheres through much of the Phanerozoic, allowing each hemisphere to record a different latitudinal diversity structure at times. In the early Palaeozoic, continents were almost entirely located in the Southern Hemisphere. By the Mesozoic and Cenozoic, however, they had drifted northwards, redistributing shallow-marine habitat area across hemispheres [47–49]. Thus, intervals with negative slopes in one hemisphere, but near-zero or positive slopes in the other, should not be interpreted as globally coherent modern-type LDGs, but as hemispherically decoupled gradients produced by the interaction between climate, tectonics, habitat availability and sampling. Palaeogeography may modify climate-related LDG slopes through its effects on the latitudinal distribution, area and continuity of shallow-marine habitats, as well as on the preservation and sampling of the fossil record [17,45]. Even when warming displaces thermal optima away from the Equator, richness may remain concentrated at low-to-middle latitudes if suitable shelf habitats are less extensive or continuous at higher latitudes. Conversely, tectonic reorganization can generate biodiversity hotspots away from the Equator by altering shelf area, coastline complexity and habitat connectivity [17]. These mechanisms may explain several deviations in our results. In the Early–Middle Ordovician, hemispherical asymmetry occurred, with negative slopes in the Northern Hemisphere and positive slopes in the Southern Hemisphere. This pattern is unlikely to represent a globally coherent modern-type LDG. Instead, it probably reflects truncated gradients owing to the absence of northern high-latitude landmasses, associated shallow-marine habitats and fossiliferous outcrops [15]. The Ordovician–Silurian decoupling therefore illustrates how palaeogeography can mediate, or even override, the expression of climate-driven LDG signals. By the Permian, a relatively even north–south alignment of the continents allowed climate to act as the primary control on the LDG, resulting in a modern-type pattern in most intervals [50]. By contrast, during the Early Triassic, shallower temperature gradients, a relatively even continental distribution and the ecological aftermath of the end-Permian mass extinction [39], including warming-driven ocean deoxygenation and aerobic habitat loss [51], led to close-to-zero or slightly positive LDG slopes. During the Mesozoic, sustained global warmth resulted in shallow latitudinal temperature gradients, while northward continental drift shifted optimal shallow-marine habitats to northern mid-latitudes [36]. This configuration may help explain both the apparent mid-latitude richness peaks during several hothouse intervals and the persistence of negative Northern Hemisphere slopes (figure 1; supplementary material, figure S4). Tropical warming may have displaced richness maxima away from the Equator without necessarily producing a positive hemisphere-wide slope when suitable habitats became less extensive at higher latitudes. Similarly, positive slopes during parts of the Palaeogene may also partly reflect the Tethyan biodiversity hotspot at low to middle northern latitudes, rather than a purely climate-driven reversal of the LDG. Because shallow-marine habitat area was not formally included as a predictor, these palaeogeographic explanations remain tentative, and their effects cannot be fully disentangled from those of changing sampling coverage.

Given that anthropogenic climate change is expected to drive global temperatures towards warmhouse or even hothouse conditions in the near future [52,53], our results imply that the present-day LDG might undergo substantial restructuring. Restructuring of the modern marine LDG is already evident, including declining tropical diversity and poleward redistribution, and these changes are projected to intensify within this century [4,54]. Deep-time climate transitions and their long-term interactions with diversity provide a key framework for predicting diversity shifts and guiding global conservation efforts in the current climate emergency.

## Supplementary Material



## Data Availability

The data, code and comprehensive README file supporting this study are available from Zenodo [55]. Supplementary material is available online [56].
