# Supplementary material for: Climate state controls the strength of the latitudinal diversity gradient
Source: Proc Biol Sci. 2026 Oct 28;293(2081):20261490. doi: 10.1098/rspb.2026.1490 (PMC13725536; doi:10.1098/rspb.2026.1490)
Supplement: Supplementary file 1 [file rspb.2026.1490.zip › rspb.2026.1490/rspb20261490_si_001.docx]

**Electronic Supplementary Material**

**Climate state controls the strength of the latitudinal diversity gradient**

Die Wen^1,2^, Lewis A. Jones^2^, Junxuan Fan^1,3*^, Philip D. Mannion^2*^

^1^School of Earth Sciences and Engineering and Frontiers Science Center for Critical Earth Material Cycling, Nanjing University, Nanjing 210023, China

^2^Department of Earth Sciences, University College London, London, WC1E 6BT, UK

^3^State Key Laboratory of Critical Earth Material Cycling and Mineral Deposits, Nanjing University, Nanjing 210023, China

*Authors for correspondence:

Junxuan Fan, e-mail: [jxfan@nju.edu.cn](mailto:jxfan@nju.edu.cn)

Philip D. Mannion, e-mail: [philipdmannion@gmail.com](mailto:philipdmannion@gmail.com)

**Journal:** *Proceedings of the Royal Society B*

**Article DOI:** 10.1098/rspb.2026.1490

**Contents:**

Supplementary methods

Figures S1 to S12

Tables S1 to S5

**Supplementary methods**

***Hemispheric sampling-structure diagnostics and their relationships with LDG slope differences***

To assess whether Northern–Southern Hemisphere differences in LDG slope were related to sampling structure, we calculated paired sampling diagnostics for stages in which both hemispheres passed quality control (QC). For each stage and hemisphere, occupied cells were counted within each absolute palaeolatitudinal bin and converted into proportional sampling profiles (figure S8). Sampling-profile dissimilarity was calculated as:

$$\boldsymbol{D}_{\boldsymbol{sampling}}=\frac{\mathbf{1}}{\mathbf{2}}\sum_{\boldsymbol{i}=\mathbf{1}}^{\boldsymbol{n}} \mid\boldsymbol{p}_{\boldsymbol{NH},\boldsymbol{i}}-\boldsymbol{p}_{\boldsymbol{SH},\boldsymbol{i}}\mid$$

where $\boldsymbol{p}_{\boldsymbol{NH},\boldsymbol{i}}$and $\boldsymbol{p}_{\boldsymbol{SH},\boldsymbol{i}}$are the proportions of occupied cells in the $\boldsymbol{i}$-th absolute palaeolatitudinal bin in the Northern and Southern Hemispheres, respectively. Values close to 0 indicate similar latitudinal sampling distributions between hemispheres, whereas higher values indicate stronger hemispheric differences in sampling structure.

We also calculated CV (k), the coefficient of variation in the number of occupied cells (k) across latitude bins, and median (k), the median number of occupied cells (k) per latitude bin. Absolute differences in these diagnostics were used to test whether sampling structure explained the magnitude of hemispheric slope discrepancies, whereas signed differences were used to test directional hemispheric offsets. Relationships were evaluated using Spearman rank correlations (table S3).

***Sensitivity analysis***

A sensitivity analysis was conducted to assess the robustness of the LDG slope estimates and the influence of key methodological parameters. Given the focus on climate–diversity relationships, we tested whether variations in spatial resolution, latitudinal binning, and richness estimation procedures substantially affected the results.

We first evaluated the impact of grid resolution by comparing LDG slopes calculated using equal-area grids with centroid spacings of 250 km, 500 km, 750 km, and 1,000 km. The corresponding figures are available in the figures_250km_0.7, figures_500km_0.7, figures_750km_0.7, and figures_1000km_0.7 folders in the Zenodo repository (https://doi.org/10.5281/zenodo.17631059). The results showed that the slope estimates remained consistent across these resolutions. However, we note that extremely large grid sizes (e.g., 1000 km) may obscure fine-scale spatial patterns, whereas smaller cells (e.g., 250 km) offer a more balanced resolution for fossil data with moderate spatial precision. Accordingly, we adopted the 250 km grid as the primary resolution for all analyses in this study.

We also tested the effect of equal-area palaeolatitudinal bin width by applying 6, 12, 18, and 36 bins. Although finer-scale bins (e.g., 36 equal-area bins) may lead to sparse data and increase noise in fossil datasets, the LDG slope estimates remained stable across all bin widths tested.

To evaluate the influence of sampling thresholds, we compared LDG slope estimates generated using occurrence-based and collection-based minimum filters. Specifically, we tested filters requiring cells to contain at least 5 or 10 occurrences, or at least 5 or 10 collections, before slope estimation (figure S10). The baseline analysis used an occurrence-minimum threshold of 5, but slope estimates were strongly correlated with those obtained under stricter occurrence- and collection-based filters.

In addition, we compared LDG slopes estimated using the per-cell resampling procedure with slopes calculated directly from all available cells. We also tested whether slopes calculated from richness values normalised within each stage differed from those calculated using raw richness values (figure S11). LDG slope estimates derived from these alternative procedures were highly correlated, indicating that the main results were not sensitive to whether slopes were estimated using resampled or all-cell data, or whether richness was normalised before slope estimation.

We further tested the effect of QC criteria used to retain stage-level hemispheric slopes. These QC scenarios combined different sampling filters, minimum cell-count thresholds per latitudinal band, adjacency requirements among effective latitudinal bands, and latitudinal-zone coverage requirements (table S4). Slopes passing each combined criterion were classified as “good” and retained for comparison. To test whether the inferred climate-state differences were also robust to QC choices, we repeated pairwise one-sided Wilcoxon rank-sum tests under each QC scenario (table S5). These tests evaluated whether LDG slopes under cooler climate states were significantly more negative than those under warmer climate states. P values were adjusted using the Benjamini–Hochberg false discovery rate correction.

Overall, sensitivity tests confirm that LDG slope estimates are robust to reasonable variations in grid spacing, latitudinal bin width, resampling strategy, richness normalisation, sampling threshold and QC criteria.

**Figure S1. Fossil collection data from the Paleobiology Database (PBDB) spanning 485.8 Ma to 0 Ma, plotted on a modern map.** (a) Present-day geographic locations of fossil collections. (b) Palaeogeographic locations of the same collections after reconstruction using the PALEOMAP model. Maps for each stage are available in the supplementary data on Zenodo: https://doi.org/10.5281/zenodo.17631059.

**Figure S2.** **Latitudinal distribution of 250 km grid cells containing fossil occurrences within equal-area palaeolatitudinal bins across geological stages.** Colours indicate the completeness of the 250 km grid cells within each equal-area palaeolatitudinal bin: green represents a complete cell and yellow represents an incomplete cell. Completeness reflects data reliability, defined as cells with ≥ 5 fossil occurrences and limited extrapolation (estimated sample size ≤ 2× observed). Panels marked ‘Bad’ indicate hemispheres with poor data quality, defined as lacking adjacent palaeolatitudinal bins in both the tropical (0–30°) and temperate (30–60°) zones, or having fewer than three bins in total. Hemispheres meeting these criteria were classified as ‘Good’ and retained for subsequent slope analyses. Sensitivity analyses using alternative bin widths (6, 12, 18, and 36 bins) are provided on Zenodo: https://doi.org/10.5281/zenodo.17631059.

**Figure S3. Temporal variation in the maximum absolute palaeolatitude of sampled occurrences in the Northern (a) and Southern (b) Hemispheres.** Filled circles denote hemisphere-stage estimates that passed the QC criteria, whereas open circles denote estimates that failed to meet these criteria. Dashed and dotted horizontal lines mark the 60° and 70° absolute palaeolatitude thresholds, respectively. Insets summarise, for each hemisphere, the percentage of time bins reaching ≥60° and ≥70°, together with the median maximum absolute palaeolatitude. Geological system: O, Ordovician; S, Silurian; D, Devonian; C, Carboniferous; P, Permian; Tr, Triassic; J, Jurassic; K, Cretaceous; Pg, Palaeogene; Ng, Neogene; Q, Quaternary. Sensitivity analyses using alternative bin widths (6, 12, 18, and 36 bins) are provided on Zenodo: https://doi.org/10.5281/zenodo.17631059.

**Figure S4. Relationship between normalised generic richness and absolute latitude across stages.** Blue circles and yellow triangles represent 250 km grid cells in the Northern and Southern Hemispheres, respectively. For each stage and hemisphere, one cell was randomly selected from each latitudinal band and the slope was calculated; this procedure was repeated 100 times. The plotted line represents the median slope estimate across resampling iterations. Grey circles and fitted lines represent hemisphere-stage estimates that did not pass the data quality criteria and are shown for reference only. Detailed figures for each stage are available on Zenodo: https://doi.org/10.5281/zenodo.17631059.

**Figure S5. Latitudinal trends in normalised generic richness across geological stages.** Coloured lines represent richness percentiles based on the 12 equal-area latitudinal bins ranging from 50% (q50) to 95% (q95), which capture variations in diversity estimates. Fitted curves show different percentile estimates. Detailed figures for each stage are available on Zenodo: https://doi.org/10.5281/zenodo.17631059.

**Figure S6. Relationship between normalised generic richness and absolute latitude across geological stages for the Northern Hemisphere.** Blue markers represent richness from 250 km grid cells. For each stage, one cell was randomly selected from each latitudinal band and the slope was calculated; this procedure was repeated 100 times. The plotted lines represent the median slope estimates across resampling iterations; light blue shadings indicate central 95% resampling intervals. Grey circles and fitted lines represent estimates that did not pass the data quality criteria and are shown for reference only. Detailed figures for each stage are available on Zenodo: https://doi.org/10.5281/zenodo.17631059.

**Figure S7.** **Relationship between normalised generic richness and absolute latitude across geological stages for the Southern Hemisphere.** Yellow markers represent richness from 250 km grid cells. For each stage, one cell was randomly selected from each latitudinal band and the slope was calculated; this procedure was repeated 100 times. The plotted lines represent the median slope estimates across resampling iterations; light yellow shadings indicate central 95% resampling intervals. Grey circles and fitted lines represent estimates that did not pass the data quality criteria and are shown for reference only. Detailed figures for each stage are available on Zenodo: https://doi.org/10.5281/zenodo.17631059.

**Figure S8. Temporal variation in Northern–Southern Hemisphere sampling-profile dissimilarity through the Phanerozoic.** Sampling-profile dissimilarity quantifies differences in the latitudinal distribution of sampled cells between hemispheres within each stage. For each hemisphere, the number of sampled cells in each absolute palaeolatitudinal band was converted into a proportional sampling profile; dissimilarity was calculated as half of the summed absolute differences between the two hemispheric profiles. Values close to zero indicate similar latitudinal sampling distributions, whereas higher values indicate stronger hemispheric differences in sampling structure. Points are connected through time to show temporal trends. Sensitivity analyses using alternative bin widths (6, 12, 18 and 36 bins) are available on Zenodo: https://doi.org/10.5281/zenodo.17631059.

**Figure S9. Relationship between Northern and Southern Hemisphere LDG slopes and interhemispheric sampling-profile dissimilarity.** (a) Comparison of LDG slopes estimated separately for the two hemispheres. The dashed diagonal line represents a 1:1 relationship, where both hemispheres have identical LDG slopes. (b) Relationship between the difference in hemispheric LDG slopes and Northern–Southern Hemisphere sampling-profile dissimilarity. Points represent paired hemisphere-stage estimates, coloured by geological era. The fitted line and grey band in (b) show the linear regression and its 95% confidence interval. Spearman’s correlation coefficient (ρ), associated P value, and sample size (n) are shown in each panel. Sensitivity analyses using alternative bin widths (6, 12, 18 and 36 bins) are available on Zenodo: https://doi.org/10.5281/zenodo.17631059.

**Figure S10.** **Comparison between baseline and filtered LDG slope estimates.** Each point represents a stage-level hemispheric LDG slope estimate, with Northern and Southern Hemisphere estimates shown in blue and orange, respectively. The baseline slope estimates are those used in the main text (occurrence-minimum threshold of 5). Filtered slopes were recalculated under stricter thresholds: occurrence ≥ 10, collection ≥5, and collection ≥10. The solid line indicates the 1:1 relationship; dashed lines mark zero slope on both axes. Spearman’s correlation coefficient and sample size are shown in each panel. Strong positive correlations indicate that the baseline resampling-based LDG slopes are robust to stricter filters. Sensitivity analyses using alternative bin widths (6, 12, 18 and 36 bins) are available on Zenodo: https://doi.org/10.5281/zenodo.17631059.

**Figure S11. Pairwise comparison of LDG slope estimates calculated using different slope-estimation procedures.** Four approaches compare the effects of resampling and richness normalisation on stage-level hemispheric slope estimates. “Direct” slopes were calculated using all available cells without resampling. “Resample” slopes were calculated by repeated random selection of one cell from each latitudinal band and using the median slope across resampling iterations. “Norm” indicates that richness values were normalised within each stage to a 0–100 scale, as in the main-text analysis. “Raw” indicates slopes calculated from unnormalised richness. Lower panels show pairwise scatterplots; diagonal panels show the distribution of slopes for each method. Upper panels show correlation coefficients for all data combined and separately for each hemisphere (blue/orange for Northern/Southern). Strong correlations indicate that the inferred LDG slope patterns are robust to whether slopes are estimated directly or through resampling, and whether richness is normalised. Sensitivity analyses using alternative bin widths (6, 12, 18 and 36 bins) are available on Zenodo: https://doi.org/10.5281/zenodo.17631059.

**Figure S12. Number of retained good LDG slope estimates under different quality control (QC) scenarios (See table S4).** Bars show the number of stage-level hemispheric LDG slopes retained as “good” under the QC scenarios defined in table S4 for the per-cell resampling OLS analysis. These scenarios combine sampling filters, minimum cell-count thresholds per latitudinal band, adjacency requirements, and latitudinal-zone coverage criteria. “T”, “Te”, and “P” indicate tropical, temperate, and polar latitudinal bands, respectively. Blue and orange bars represent Northern and Southern Hemisphere estimates. Higher values indicate that more slopes passed the specified QC criteria. Sensitivity analyses using alternative bin widths (6, 12, 18 and 36 bins) are available on Zenodo: https://doi.org/10.5281/zenodo.17631059.

**Table S1. Pairwise Wilcoxon rank-sum one-sided tests for comparisons of LDG slopes among different climate states.** Each directional test evaluates whether the slopes under a cooler climate state are significantly more negative than those under a warmer climate state. “Analysis scope” indicates whether comparisons used slopes from both hemispheres combined (“Combined”) or separately for the Northern and Southern Hemispheres. The table reports median differences (group 1 versus group 2), W statistics, nominal *p* values and *p*-adjusted values. Adjusted *p* values were calculated using the Benjamini–Hochberg false discovery rate correction. Asterisks denote significance: *p* < 0.05 (*), *p* < 0.01 (**), and *p* < 0.001 (***).

| **Analysis scope** | **Group 1** | **Group 2** | **Median difference** | **W statistics** | ***p* value** | ***p*-adjusted value** |
| --- | --- | --- | --- | --- | --- | --- |
| **Combined** | **Coldhouse** | **Transitional** | -0.336 | 126*** | 0.000 | 0.001 |
| **Combined** | **Coldhouse** | **Warmhouse** | -0.274 | 190*** | 0.000 | 0.001 |
| **Combined** | **Coolhouse** | **Warmhouse** | -0.320 | 151*** | 0.000 | 0.001 |
| **Combined** | **Coldhouse** | **Hothouse** | -0.168 | 224** | 0.002 | 0.005 |
| **Combined** | **Coolhouse** | **Transitional** | -0.152 | 261* | 0.007 | 0.014 |
| **Combined** | **Coolhouse** | **Hothouse** | -0.106 | 333* | 0.013 | 0.022 |
| **Combined** | **Coldhouse** | **Coolhouse** | -0.168 | 267 | 0.054 | 0.077 |
| **Combined** | **Transitional** | **Warmhouse** | -0.062 | 361 | 0.106 | 0.132 |
| **Combined** | **Transitional** | **Hothouse** | -0.046 | 411 | 0.232 | 0.258 |
| **Combined** | **Warmhouse** | **Hothouse** | -0.016 | 347 | 0.304 | 0.304 |
| **Northern** | **Coldhouse** | **Warmhouse** | -0.402 | 26** | 0.001 | 0.008 |
| **Northern** | **Coldhouse** | **Transitional** | -0.382 | 37** | 0.002 | 0.008 |
| **Northern** | **Coolhouse** | **Warmhouse** | -0.226 | 45** | 0.003 | 0.008 |
| **Northern** | **Coldhouse** | **Hothouse** | -0.273 | 21** | 0.003 | 0.008 |
| **Northern** | **Coolhouse** | **Transitional** | -0.206 | 65* | 0.010 | 0.019 |
| **Northern** | **Coolhouse** | **Hothouse** | -0.096 | 48 | 0.039 | 0.065 |
| **Northern** | **Coldhouse** | **Coolhouse** | -0.176 | 69 | 0.159 | 0.227 |
| **Northern** | **Transitional** | **Warmhouse** | -0.020 | 113 | 0.299 | 0.373 |
| **Northern** | **Transitional** | **Hothouse** | 0.110 | 111 | 0.801 | 0.862 |
| **Northern** | **Warmhouse** | **Hothouse** | 0.130 | 103 | 0.862 | 0.862 |
| **Southern** | **Coldhouse** | **Hothouse** | -0.375 | 40* | 0.005 | 0.050 |
| **Southern** | **Transitional** | **Hothouse** | -0.155 | 68 | 0.012 | 0.062 |
| **Southern** | **Coolhouse** | **Hothouse** | -0.181 | 73 | 0.033 | 0.092 |
| **Southern** | **Coldhouse** | **Transitional** | -0.220 | 57 | 0.037 | 0.092 |
| **Southern** | **Warmhouse** | **Hothouse** | -0.084 | 68 | 0.060 | 0.101 |
| **Southern** | **Coldhouse** | **Warmhouse** | -0.291 | 49 | 0.061 | 0.101 |
| **Southern** | **Coldhouse** | **Coolhouse** | -0.194 | 65 | 0.116 | 0.166 |
| **Southern** | **Coolhouse** | **Transitional** | -0.026 | 102 | 0.245 | 0.272 |
| **Southern** | **Coolhouse** | **Warmhouse** | -0.097 | 82 | 0.245 | 0.272 |
| **Southern** | **Transitional** | **Warmhouse** | -0.071 | 97 | 0.388 | 0.388 |

**Table S2. Time bin definitions including names, temporal boundaries, and corresponding climate states.** Climate state classification follows Judd et al., 2024 [1].

| **Interval name** | **Max (Ma)** | **Mid (Ma)** | **Min (Ma)** | **Abbreviation** | **System** | **Climate state** |
| --- | --- | --- | --- | --- | --- | --- |
| Tremadocian | 486.85 | 481.975 | 477.1 | Tre | Ordovician | Hothouse |
| Floian | 477.1 | 474.2 | 471.3 | Flo | Ordovician | Hothouse |
| Dapingian | 471.3 | 470.35 | 469.4 | Dap | Ordovician | Hothouse |
| Darriwilian | 469.4 | 463.8 | 458.2 | Dar | Ordovician | Hothouse |
| Sandbian | 458.2 | 455.5 | 452.8 | Sbd | Ordovician | Warmhouse |
| Katian | 452.8 | 449 | 445.2 | Kat | Ordovician | Warmhouse |
| Hirnantian | 445.2 | 444.15 | 443.1 | Hir | Ordovician | Coolhouse |
| Rhuddanian | 443.1 | 441.8 | 440.5 | Rhu | Silurian | Coolhouse |
| Aeronian | 440.5 | 439.55 | 438.6 | Aer | Silurian | Coolhouse |
| Telychian | 438.6 | 435.75 | 432.9 | Tel | Silurian | Transitional |
| Sheinwoodian | 432.9 | 431.75 | 430.6 | She | Silurian | Coolhouse |
| Homerian | 430.6 | 428.65 | 426.7 | Hom | Silurian | Coolhouse |
| Gorstian | 426.7 | 425.85 | 425 | Gor | Silurian | Warmhouse |
| Ludfordian | 425 | 423.85 | 422.7 | Lud | Silurian | Hothouse |
| Pridoli | 422.7 | 421.16 | 419.62 | Pri | Silurian | Hothouse |
| Lochkovian | 419.62 | 416.32 | 413.02 | Loc | Devonian | Hothouse |
| Pragian | 413.02 | 411.82 | 410.62 | Pra | Devonian | Hothouse |
| Emsian | 410.62 | 402.045 | 393.47 | Ems | Devonian | Transitional |
| Eifelian | 393.47 | 390.71 | 387.95 | Eif | Devonian | Transitional |
| Givetian | 387.95 | 385.13 | 382.31 | Giv | Devonian | Warmhouse |
| Frasnian | 382.31 | 377.23 | 372.15 | Fra | Devonian | Warmhouse |
| Famennian | 372.15 | 365.505 | 358.86 | Fam | Devonian | Transitional |
| Tournaisian | 358.86 | 352.78 | 346.7 | Tou | Carboniferous | Coolhouse |
| Visean | 346.7 | 338.5 | 330.3 | Vis | Carboniferous | Coldhouse |
| Serpukhovian | 330.3 | 326.85 | 323.4 | Ser | Carboniferous | Coldhouse |
| Bashkirian | 323.4 | 319.3 | 315.2 | Bas | Carboniferous | Coldhouse |
| Moscovian | 315.2 | 311.1 | 307 | Mos | Carboniferous | Coldhouse |
| Kasimovian | 307 | 305.35 | 303.7 | Kas | Carboniferous | Coldhouse |
| Gzhelian | 303.7 | 301.3 | 298.9 | Gzh | Carboniferous | Coldhouse |
| Asselian | 298.9 | 296.21 | 293.52 | Ass | Permian | Coolhouse |
| Sakmarian | 293.52 | 291.81 | 290.1 | Sak | Permian | Coolhouse |
| Artinskian | 290.1 | 286.7 | 283.3 | Art | Permian | Coolhouse |
| Kungurian | 283.3 | 278.85 | 274.4 | Kun | Permian | Coolhouse |
| Roadian | 274.4 | 270.65 | 266.9 | Roa | Permian | Coldhouse |
| Wordian | 266.9 | 265.59 | 264.28 | Wor | Permian | Coolhouse |
| Capitanian | 264.28 | 261.895 | 259.51 | Cap | Permian | Coolhouse |
| Wuchiapingian | 259.51 | 256.825 | 254.14 | Wuc | Permian | Coolhouse |
| Changhsingian | 254.14 | 253.021 | 251.902 | Chg | Permian | Coldhouse |
| Induan | 251.902 | 250.901 | 249.9 | Ind | Triassic | Hothouse |
| Olenekian | 249.9 | 248.3 | 246.7 | Ole | Triassic | Warmhouse |
| Anisian | 246.7 | 244.082 | 241.464 | Ani | Triassic | Warmhouse |
| Ladinian | 241.464 | 239.232 | 237 | Lad | Triassic | Coolhouse |
| Carnian | 237 | 232.15 | 227.3 | Car | Triassic | Transitional |
| Norian | 227.3 | 216.5 | 205.7 | Nor | Triassic | Transitional |
| Rhaetian | 205.7 | 203.55 | 201.4 | Rha | Triassic | Coolhouse |
| Hettangian | 201.4 | 200.45 | 199.5 | Het | Jurassic | Transitional |
| Sinemurian | 199.5 | 196.2 | 192.9 | Sin | Jurassic | Transitional |
| Pliensbachian | 192.9 | 188.55 | 184.2 | Plb | Jurassic | Transitional |
| Toarcian | 184.2 | 179.45 | 174.7 | Toa | Jurassic | Warmhouse |
| Aalenian | 174.7 | 172.8 | 170.9 | Aal | Jurassic | Transitional |
| Bajocian | 170.9 | 169.55 | 168.2 | Baj | Jurassic | Coolhouse |
| Bathonian | 168.2 | 166.75 | 165.3 | Bat | Jurassic | Coldhouse |
| Callovian | 165.3 | 163.4 | 161.5 | Cal | Jurassic | Coolhouse |
| Oxfordian | 161.5 | 158.15 | 154.8 | Oxf | Jurassic | Transitional |
| Kimmeridgian | 154.8 | 152 | 149.2 | Kim | Jurassic | Transitional |
| Tithonian | 149.2 | 146.15 | 143.1 | Tit | Jurassic | Warmhouse |
| Berriasian | 143.1 | 140.075 | 137.05 | Ber | Cretaceous | Warmhouse |
| Valanginian | 137.05 | 134.825 | 132.6 | Val | Cretaceous | Warmhouse |
| Hauterivian | 132.6 | 129.185 | 125.77 | Hau | Cretaceous | Warmhouse |
| Barremian | 125.77 | 123.585 | 121.4 | Bar | Cretaceous | Hothouse |
| Aptian | 121.4 | 117.3 | 113.2 | Apt | Cretaceous | Hothouse |
| Albian | 113.2 | 106.85 | 100.5 | Alb | Cretaceous | Warmhouse |
| Cenomanian | 100.5 | 97.2 | 93.9 | Cen | Cretaceous | Hothouse |
| Turonian | 93.9 | 91.85 | 89.8 | Tur | Cretaceous | Hothouse |
| Coniacian | 89.8 | 87.75 | 85.7 | Con | Cretaceous | Hothouse |
| Santonian | 85.7 | 84.65 | 83.6 | San | Cretaceous | Hothouse |
| Campanian | 83.6 | 77.9 | 72.2 | Cam | Cretaceous | Warmhouse |
| Maastrichtian | 72.2 | 69.1 | 66 | Maa | Cretaceous | Transitional |
| Danian | 66 | 63.83 | 61.66 | Dan | Palaeogene | Hothouse |
| Selandian | 61.66 | 60.45 | 59.24 | Se | Palaeogene | Hothouse |
| Thanetian | 59.24 | 57.62 | 56 | Th | Palaeogene | Warmhouse |
| Ypresian | 56 | 52.035 | 48.07 | Ypr | Palaeogene | Hothouse |
| Lutetian | 48.07 | 44.55 | 41.03 | Lut | Palaeogene | Warmhouse |
| Bartonian | 41.03 | 39.37 | 37.71 | Brt | Palaeogene | Warmhouse |
| Priabonian | 37.71 | 35.805 | 33.9 | Prb | Palaeogene | Warmhouse |
| Rupelian | 33.9 | 30.6 | 27.3 | Rup | Palaeogene | Transitional |
| Chattian | 27.3 | 25.17 | 23.04 | Cht | Palaeogene | Transitional |
| Aquitanian | 23.04 | 21.745 | 20.45 | Aqu | Neogene | Coolhouse |
| Burdigalian | 20.45 | 18.215 | 15.98 | Bur | Neogene | Transitional |
| Langhian | 15.98 | 14.9 | 13.82 | Lan | Neogene | Transitional |
| Serravallian | 13.82 | 12.725 | 11.63 | Ser | Neogene | Transitional |
| Tortonian | 11.63 | 9.438 | 7.246 | Tor | Neogene | Coolhouse |
| Messinian | 7.246 | 6.2895 | 5.333 | Mes | Neogene | Coldhouse |
| Zanclean | 5.333 | 4.4665 | 3.6 | Zan | Neogene | Coldhouse |
| Piacenzian | 3.6 | 3.09 | 2.58 | Pia | Neogene | Coldhouse |
| Pleistocene | 2.58 | 1.29585 | 0.0117 | Ple | Quaternary | Coldhouse |
| Holocene | 0.0117 | 0.00585 | 0 | Hol | Quaternary | Coldhouse |

**Table S3. Paired hemispheric slope and sampling-structure diagnostics.** Spearman rank correlations testing relationships between Northern and Southern Hemisphere LDG slope estimates and selected sampling diagnostics for stages in which both hemispheres passed quality control. |NH-SH slope| represents absolute slope differences used to assess whether sampling differences were associated with the magnitude of hemispheric slope discrepancies. NH-SH slope represents signed slope differences used to assess directional hemispheric offsets. Sampling-profile dissimilarity measures differences in the proportional distribution of occupied cells across absolute-latitude bins between hemispheres; cv (k) difference represents latitudinal-bin sampling unevenness; and median (k) asymmetry represents the median number of occupied cells per latitudinal bin.

| test | n | estimate | P value |
| --- | --- | --- | --- |
| NH slope vs SH slope | 59 | 0.246 | 0.061 |
| \|NH-SH slope\| vs sampling-profile dissimilarity | 59 | 0.199 | 0.130 |
| \|NH-SH slope\| vs absolute cv (k) difference | 59 | 0.016 | 0.905 |
| \|NH-SH slope\| vs absolute median (k) asymmetry | 59 | -0.336 | 0.009 |
| NH-SH slope vs sampling-profile dissimilarity | 59 | 0.116 | 0.382 |
| NH-SH slope vs signed cv (k) difference | 59 | 0.207 | 0.115 |
| NH-SH slope vs signed median (k) asymmetry | 59 | 0.099 | 0.456 |

**Table S4. QC scenarios used to assess the robustness of LDG slope estimates.**

| **Quality control dimension** | **Tested levels** | **Description** |
| --- | --- | --- |
| Sampling filter | occurrence ≥ 5; occurrence ≥ 10; collection ≥ 5; collection ≥ 10 | Minimum sampling requirement used to retain cells before slope estimation. |
| Minimum cells per latitudinal band | *k* = 1, 2, 3 | Effective latitudinal bands were first defined by the minimum cell-count threshold for each hemisphere–stage combination. Continuity was then assessed within 0–60° absolute palaeolatitude, requiring at least three effective bands, with at least two adjacent. |
| Latitudinal coverage requirement | tropical + temperate; tropical + temperate + polar | After applying the minimum cell-count and adjacency filters, effective bands were checked for zone coverage. The less strict scenario required tropical–temperate coverage, whereas the stricter scenario additionally required polar coverage. |

**Table S5. Pairwise one-sided Wilcoxon rank-sum tests comparing LDG slopes among climate states under different QC scenarios (See table S4).** Each directional test evaluates whether the slopes under a cooler climate state are significantly lesser than those under a warmer climate state. “Analysis scope” indicates whether comparisons used slopes from both hemispheres combined or separately for the Northern and Southern Hemispheres. The table reports the sample sizes for group 1 (n1) and group 2 (n2), median differences between groups, W statistics, nominal p values, and Benjamini–Hochberg false-discovery-rate adjusted p values.

| **Analysis scope** | **QC Scenario** | **Group 1** | **Group 2** | **n1** | **n2** | **Median difference** | **W statistics** | **p value** | **p-adjusted value** |
| --- | --- | --- | --- | --- | --- | --- | --- | --- | --- |
| Combined | occurrence5_k1_tropical_temperate | Coldhouse | Hothouse | 24 | 27 | -0.34 | 126 | 0.000 | 0.001 |
| Combined | occurrence5_k1_tropical_temperate | Coldhouse | Transitional | 24 | 33 | -0.27 | 190 | 0.000 | 0.001 |
| Combined | occurrence5_k1_tropical_temperate | Coldhouse | Warmhouse | 24 | 28 | -0.32 | 151 | 0.000 | 0.001 |
| Combined | occurrence5_k1_tropical_temperate | Coolhouse | Hothouse | 30 | 27 | -0.17 | 224 | 0.002 | 0.005 |
| Combined | occurrence5_k1_tropical_temperate | Coolhouse | Warmhouse | 30 | 28 | -0.15 | 261 | 0.007 | 0.014 |
| Combined | occurrence5_k1_tropical_temperate | Coolhouse | Transitional | 30 | 33 | -0.11 | 333 | 0.013 | 0.022 |
| Combined | occurrence5_k1_tropical_temperate | Coldhouse | Coolhouse | 24 | 30 | -0.17 | 267 | 0.054 | 0.077 |
| Combined | occurrence5_k1_tropical_temperate | Transitional | Hothouse | 33 | 27 | -0.06 | 361 | 0.106 | 0.132 |
| Combined | occurrence5_k1_tropical_temperate | Transitional | Warmhouse | 33 | 28 | -0.05 | 411 | 0.232 | 0.258 |
| Combined | occurrence5_k1_tropical_temperate | Warmhouse | Hothouse | 28 | 27 | -0.02 | 347 | 0.304 | 0.304 |
| Combined | occurrence5_k1_tropical_temperate_polar | Coldhouse | Hothouse | 12 | 20 | -0.29 | 33 | 0.000 | 0.004 |
| Combined | occurrence5_k1_tropical_temperate_polar | Coldhouse | Transitional | 12 | 25 | -0.24 | 57 | 0.001 | 0.006 |
| Combined | occurrence5_k1_tropical_temperate_polar | Coolhouse | Hothouse | 23 | 20 | -0.20 | 109 | 0.002 | 0.006 |
| Combined | occurrence5_k1_tropical_temperate_polar | Coldhouse | Warmhouse | 12 | 23 | -0.27 | 56 | 0.002 | 0.006 |
| Combined | occurrence5_k1_tropical_temperate_polar | Coolhouse | Warmhouse | 23 | 23 | -0.18 | 153 | 0.007 | 0.015 |
| Combined | occurrence5_k1_tropical_temperate_polar | Coolhouse | Transitional | 23 | 25 | -0.14 | 184 | 0.017 | 0.028 |
| Combined | occurrence5_k1_tropical_temperate_polar | Transitional | Hothouse | 25 | 20 | -0.06 | 187 | 0.077 | 0.110 |
| Combined | occurrence5_k1_tropical_temperate_polar | Coldhouse | Coolhouse | 12 | 23 | -0.10 | 107 | 0.145 | 0.181 |
| Combined | occurrence5_k1_tropical_temperate_polar | Transitional | Warmhouse | 25 | 23 | -0.04 | 255 | 0.254 | 0.254 |
| Combined | occurrence5_k1_tropical_temperate_polar | Warmhouse | Hothouse | 23 | 20 | -0.02 | 201 | 0.244 | 0.254 |
| Combined | occurrence5_k2_tropical_temperate | Coldhouse | Transitional | 17 | 22 | -0.22 | 110 | 0.015 | 0.053 |
| Combined | occurrence5_k2_tropical_temperate | Coldhouse | Warmhouse | 17 | 20 | -0.24 | 103 | 0.021 | 0.053 |
| Combined | occurrence5_k2_tropical_temperate | Coldhouse | Hothouse | 17 | 20 | -0.27 | 86 | 0.005 | 0.053 |
| Combined | occurrence5_k2_tropical_temperate | Coolhouse | Hothouse | 26 | 20 | -0.10 | 163 | 0.016 | 0.053 |
| Combined | occurrence5_k2_tropical_temperate | Coolhouse | Transitional | 26 | 22 | -0.05 | 218 | 0.081 | 0.138 |
| Combined | occurrence5_k2_tropical_temperate | Coolhouse | Warmhouse | 26 | 20 | -0.06 | 197 | 0.083 | 0.138 |
| Combined | occurrence5_k2_tropical_temperate | Coldhouse | Coolhouse | 17 | 26 | -0.17 | 183 | 0.176 | 0.220 |
| Combined | occurrence5_k2_tropical_temperate | Transitional | Hothouse | 22 | 20 | -0.05 | 181 | 0.166 | 0.220 |
| Combined | occurrence5_k2_tropical_temperate | Warmhouse | Hothouse | 20 | 20 | -0.03 | 175 | 0.254 | 0.282 |
| Combined | occurrence5_k2_tropical_temperate | Transitional | Warmhouse | 22 | 20 | -0.02 | 212 | 0.425 | 0.425 |
| Combined | occurrence5_k2_tropical_temperate_polar | Coldhouse | Transitional | 8 | 12 | -0.22 | 22 | 0.025 | 0.066 |
| Combined | occurrence5_k2_tropical_temperate_polar | Coldhouse | Warmhouse | 8 | 15 | -0.24 | 31 | 0.033 | 0.066 |
| Combined | occurrence5_k2_tropical_temperate_polar | Coldhouse | Hothouse | 8 | 13 | -0.29 | 19 | 0.009 | 0.066 |
| Combined | occurrence5_k2_tropical_temperate_polar | Coolhouse | Warmhouse | 17 | 15 | -0.08 | 78 | 0.032 | 0.066 |
| Combined | occurrence5_k2_tropical_temperate_polar | Coolhouse | Hothouse | 17 | 13 | -0.13 | 58 | 0.015 | 0.066 |
| Combined | occurrence5_k2_tropical_temperate_polar | Transitional | Hothouse | 12 | 13 | -0.07 | 55 | 0.111 | 0.184 |
| Combined | occurrence5_k2_tropical_temperate_polar | Coolhouse | Transitional | 17 | 12 | -0.05 | 77 | 0.139 | 0.199 |
| Combined | occurrence5_k2_tropical_temperate_polar | Transitional | Warmhouse | 12 | 15 | -0.03 | 76 | 0.255 | 0.319 |
| Combined | occurrence5_k2_tropical_temperate_polar | Coldhouse | Coolhouse | 8 | 17 | -0.16 | 58 | 0.290 | 0.322 |
| Combined | occurrence5_k2_tropical_temperate_polar | Warmhouse | Hothouse | 15 | 13 | -0.05 | 92 | 0.409 | 0.409 |
| Combined | occurrence5_k3_tropical_temperate | Coldhouse | Hothouse | 11 | 14 | -0.31 | 29 | 0.005 | 0.047 |
| Combined | occurrence5_k3_tropical_temperate | Coldhouse | Transitional | 11 | 16 | -0.25 | 51 | 0.036 | 0.115 |
| Combined | occurrence5_k3_tropical_temperate | Coolhouse | Hothouse | 13 | 14 | -0.16 | 50 | 0.025 | 0.115 |
| Combined | occurrence5_k3_tropical_temperate | Warmhouse | Hothouse | 16 | 14 | -0.11 | 71 | 0.046 | 0.115 |
| Combined | occurrence5_k3_tropical_temperate | Coldhouse | Warmhouse | 11 | 16 | -0.20 | 57 | 0.066 | 0.117 |
| Combined | occurrence5_k3_tropical_temperate | Transitional | Hothouse | 16 | 14 | -0.07 | 76 | 0.070 | 0.117 |
| Combined | occurrence5_k3_tropical_temperate | Coolhouse | Transitional | 13 | 16 | -0.09 | 78 | 0.132 | 0.188 |
| Combined | occurrence5_k3_tropical_temperate | Coolhouse | Warmhouse | 13 | 16 | -0.05 | 84 | 0.196 | 0.245 |
| Combined | occurrence5_k3_tropical_temperate | Coldhouse | Coolhouse | 11 | 13 | -0.16 | 64 | 0.343 | 0.381 |
| Combined | occurrence5_k3_tropical_temperate | Transitional | Warmhouse | 16 | 16 | 0.04 | 140 | 0.681 | 0.681 |
| Combined | occurrence5_k3_tropical_temperate_polar | Coldhouse | Hothouse | 5 | 8 | -0.29 | 1 | 0.003 | 0.034 |
| Combined | occurrence5_k3_tropical_temperate_polar | Coldhouse | Transitional | 5 | 8 | -0.22 | 6 | 0.024 | 0.070 |
| Combined | occurrence5_k3_tropical_temperate_polar | Coolhouse | Hothouse | 7 | 8 | -0.14 | 11 | 0.028 | 0.070 |
| Combined | occurrence5_k3_tropical_temperate_polar | Transitional | Hothouse | 8 | 8 | -0.07 | 13 | 0.026 | 0.070 |
| Combined | occurrence5_k3_tropical_temperate_polar | Coldhouse | Warmhouse | 5 | 10 | -0.20 | 10 | 0.038 | 0.076 |
| Combined | occurrence5_k3_tropical_temperate_polar | Warmhouse | Hothouse | 10 | 8 | -0.09 | 21 | 0.050 | 0.084 |
| Combined | occurrence5_k3_tropical_temperate_polar | Coolhouse | Warmhouse | 7 | 10 | -0.05 | 25 | 0.177 | 0.253 |
| Combined | occurrence5_k3_tropical_temperate_polar | Coolhouse | Transitional | 7 | 8 | -0.07 | 21 | 0.226 | 0.282 |
| Combined | occurrence5_k3_tropical_temperate_polar | Coldhouse | Coolhouse | 5 | 7 | -0.16 | 13 | 0.258 | 0.287 |
| Combined | occurrence5_k3_tropical_temperate_polar | Transitional | Warmhouse | 8 | 10 | 0.02 | 42 | 0.588 | 0.588 |
| Combined | occurrence10_k1_tropical_temperate | Coldhouse | Warmhouse | 23 | 26 | -0.50 | 104 | 0.000 | 0.000 |
| Combined | occurrence10_k1_tropical_temperate | Coldhouse | Hothouse | 23 | 27 | -0.47 | 119 | 0.000 | 0.001 |
| Combined | occurrence10_k1_tropical_temperate | Coldhouse | Transitional | 23 | 32 | -0.40 | 173 | 0.000 | 0.002 |
| Combined | occurrence10_k1_tropical_temperate | Coolhouse | Warmhouse | 30 | 26 | -0.26 | 234 | 0.005 | 0.011 |
| Combined | occurrence10_k1_tropical_temperate | Coolhouse | Hothouse | 30 | 27 | -0.22 | 244 | 0.005 | 0.011 |
| Combined | occurrence10_k1_tropical_temperate | Coolhouse | Transitional | 30 | 32 | -0.16 | 341 | 0.026 | 0.043 |
| Combined | occurrence10_k1_tropical_temperate | Coldhouse | Coolhouse | 23 | 30 | -0.24 | 241 | 0.032 | 0.045 |
| Combined | occurrence10_k1_tropical_temperate | Transitional | Warmhouse | 32 | 26 | -0.10 | 332 | 0.096 | 0.120 |
| Combined | occurrence10_k1_tropical_temperate | Transitional | Hothouse | 32 | 27 | -0.07 | 352 | 0.113 | 0.126 |
| Combined | occurrence10_k1_tropical_temperate | Warmhouse | Hothouse | 26 | 27 | 0.03 | 343 | 0.447 | 0.447 |
| Combined | occurrence10_k1_tropical_temperate_polar | Coldhouse | Warmhouse | 10 | 22 | -0.30 | 44 | 0.004 | 0.026 |
| Combined | occurrence10_k1_tropical_temperate_polar | Coldhouse | Hothouse | 10 | 18 | -0.33 | 36 | 0.005 | 0.026 |
| Combined | occurrence10_k1_tropical_temperate_polar | Coldhouse | Transitional | 10 | 24 | -0.20 | 62 | 0.015 | 0.040 |
| Combined | occurrence10_k1_tropical_temperate_polar | Coolhouse | Hothouse | 20 | 18 | -0.23 | 106 | 0.016 | 0.040 |
| Combined | occurrence10_k1_tropical_temperate_polar | Coolhouse | Warmhouse | 20 | 22 | -0.20 | 140 | 0.023 | 0.045 |
| Combined | occurrence10_k1_tropical_temperate_polar | Transitional | Hothouse | 24 | 18 | -0.13 | 154 | 0.059 | 0.098 |
| Combined | occurrence10_k1_tropical_temperate_polar | Transitional | Warmhouse | 24 | 22 | -0.10 | 197 | 0.072 | 0.103 |
| Combined | occurrence10_k1_tropical_temperate_polar | Coolhouse | Transitional | 20 | 24 | -0.10 | 189 | 0.117 | 0.146 |
| Combined | occurrence10_k1_tropical_temperate_polar | Coldhouse | Coolhouse | 10 | 20 | -0.10 | 76 | 0.151 | 0.167 |
| Combined | occurrence10_k1_tropical_temperate_polar | Warmhouse | Hothouse | 22 | 18 | -0.03 | 174 | 0.261 | 0.261 |
| Combined | occurrence10_k2_tropical_temperate | Coldhouse | Warmhouse | 16 | 19 | -0.19 | 84 | 0.013 | 0.042 |
| Combined | occurrence10_k2_tropical_temperate | Coldhouse | Hothouse | 16 | 20 | -0.29 | 80 | 0.006 | 0.042 |
| Combined | occurrence10_k2_tropical_temperate | Coolhouse | Hothouse | 23 | 20 | -0.24 | 137 | 0.012 | 0.042 |
| Combined | occurrence10_k2_tropical_temperate | Coldhouse | Transitional | 16 | 20 | -0.19 | 106 | 0.044 | 0.098 |
| Combined | occurrence10_k2_tropical_temperate | Coolhouse | Warmhouse | 23 | 19 | -0.13 | 156 | 0.059 | 0.098 |
| Combined | occurrence10_k2_tropical_temperate | Transitional | Hothouse | 20 | 20 | -0.11 | 140 | 0.054 | 0.098 |
| Combined | occurrence10_k2_tropical_temperate | Coolhouse | Transitional | 23 | 20 | -0.13 | 179 | 0.109 | 0.156 |
| Combined | occurrence10_k2_tropical_temperate | Transitional | Warmhouse | 20 | 19 | 0.00 | 160 | 0.204 | 0.254 |
| Combined | occurrence10_k2_tropical_temperate | Coldhouse | Coolhouse | 16 | 23 | -0.05 | 163 | 0.279 | 0.279 |
| Combined | occurrence10_k2_tropical_temperate | Warmhouse | Hothouse | 19 | 20 | -0.11 | 168 | 0.273 | 0.279 |
| Combined | occurrence10_k2_tropical_temperate_polar | Coldhouse | Hothouse | 7 | 10 | -0.32 | 11 | 0.011 | 0.055 |
| Combined | occurrence10_k2_tropical_temperate_polar | Coolhouse | Hothouse | 12 | 10 | -0.26 | 24 | 0.010 | 0.055 |
| Combined | occurrence10_k2_tropical_temperate_polar | Transitional | Hothouse | 10 | 10 | -0.15 | 25 | 0.032 | 0.107 |
| Combined | occurrence10_k2_tropical_temperate_polar | Coldhouse | Warmhouse | 7 | 12 | -0.16 | 24 | 0.070 | 0.174 |
| Combined | occurrence10_k2_tropical_temperate_polar | Coldhouse | Transitional | 7 | 10 | -0.17 | 21 | 0.094 | 0.188 |
| Combined | occurrence10_k2_tropical_temperate_polar | Coolhouse | Warmhouse | 12 | 12 | -0.10 | 53 | 0.143 | 0.204 |
| Combined | occurrence10_k2_tropical_temperate_polar | Warmhouse | Hothouse | 12 | 10 | -0.16 | 43 | 0.138 | 0.204 |
| Combined | occurrence10_k2_tropical_temperate_polar | Coolhouse | Transitional | 12 | 10 | -0.11 | 49 | 0.244 | 0.305 |
| Combined | occurrence10_k2_tropical_temperate_polar | Coldhouse | Coolhouse | 7 | 12 | -0.06 | 36 | 0.321 | 0.321 |
| Combined | occurrence10_k2_tropical_temperate_polar | Transitional | Warmhouse | 10 | 12 | 0.01 | 52 | 0.310 | 0.321 |
| Combined | occurrence10_k3_tropical_temperate | Coldhouse | Warmhouse | 9 | 14 | -0.15 | 27 | 0.013 | 0.063 |
| Combined | occurrence10_k3_tropical_temperate | Coldhouse | Hothouse | 9 | 11 | -0.34 | 18 | 0.009 | 0.063 |
| Combined | occurrence10_k3_tropical_temperate | Coldhouse | Transitional | 9 | 15 | -0.25 | 33 | 0.021 | 0.071 |
| Combined | occurrence10_k3_tropical_temperate | Coolhouse | Hothouse | 11 | 11 | -0.22 | 31 | 0.028 | 0.071 |
| Combined | occurrence10_k3_tropical_temperate | Coolhouse | Transitional | 11 | 15 | -0.14 | 48 | 0.039 | 0.078 |
| Combined | occurrence10_k3_tropical_temperate | Coolhouse | Warmhouse | 11 | 14 | -0.04 | 56 | 0.131 | 0.172 |
| Combined | occurrence10_k3_tropical_temperate | Transitional | Hothouse | 15 | 11 | -0.09 | 61 | 0.138 | 0.172 |
| Combined | occurrence10_k3_tropical_temperate | Warmhouse | Hothouse | 14 | 11 | -0.19 | 56 | 0.131 | 0.172 |
| Combined | occurrence10_k3_tropical_temperate | Coldhouse | Coolhouse | 9 | 11 | -0.11 | 39 | 0.224 | 0.249 |
| Combined | occurrence10_k3_tropical_temperate | Transitional | Warmhouse | 15 | 14 | 0.10 | 108 | 0.561 | 0.561 |
| Combined | occurrence10_k3_tropical_temperate_polar | Coldhouse | Transitional | 4 | 7 | -0.23 | 3 | 0.024 | 0.079 |
| Combined | occurrence10_k3_tropical_temperate_polar | Coldhouse | Warmhouse | 4 | 7 | -0.19 | 3 | 0.024 | 0.079 |
| Combined | occurrence10_k3_tropical_temperate_polar | Coldhouse | Hothouse | 4 | 6 | -0.30 | 2 | 0.021 | 0.079 |
| Combined | occurrence10_k3_tropical_temperate_polar | Coldhouse | Coolhouse | 4 | 4 | -0.19 | 3 | 0.097 | 0.242 |
| Combined | occurrence10_k3_tropical_temperate_polar | Coolhouse | Hothouse | 4 | 6 | -0.11 | 7 | 0.169 | 0.281 |
| Combined | occurrence10_k3_tropical_temperate_polar | Transitional | Hothouse | 7 | 6 | -0.06 | 13 | 0.142 | 0.281 |
| Combined | occurrence10_k3_tropical_temperate_polar | Coolhouse | Warmhouse | 4 | 7 | -0.01 | 10 | 0.254 | 0.325 |
| Combined | occurrence10_k3_tropical_temperate_polar | Warmhouse | Hothouse | 7 | 6 | -0.10 | 16 | 0.260 | 0.325 |
| Combined | occurrence10_k3_tropical_temperate_polar | Coolhouse | Transitional | 4 | 7 | -0.05 | 11 | 0.318 | 0.354 |
| Combined | occurrence10_k3_tropical_temperate_polar | Transitional | Warmhouse | 7 | 7 | 0.04 | 23 | 0.449 | 0.449 |
| Combined | collection5_k1_tropical_temperate | Coldhouse | Warmhouse | 23 | 25 | -0.50 | 94 | 0.000 | 0.000 |
| Combined | collection5_k1_tropical_temperate | Coldhouse | Hothouse | 23 | 23 | -0.43 | 98 | 0.000 | 0.001 |
| Combined | collection5_k1_tropical_temperate | Coldhouse | Transitional | 23 | 31 | -0.44 | 163 | 0.000 | 0.001 |
| Combined | collection5_k1_tropical_temperate | Coolhouse | Warmhouse | 29 | 25 | -0.22 | 195 | 0.002 | 0.004 |
| Combined | collection5_k1_tropical_temperate | Coolhouse | Hothouse | 29 | 23 | -0.15 | 177 | 0.002 | 0.004 |
| Combined | collection5_k1_tropical_temperate | Coolhouse | Transitional | 29 | 31 | -0.15 | 306 | 0.017 | 0.029 |
| Combined | collection5_k1_tropical_temperate | Coldhouse | Coolhouse | 23 | 29 | -0.28 | 240 | 0.043 | 0.062 |
| Combined | collection5_k1_tropical_temperate | Transitional | Warmhouse | 31 | 25 | -0.07 | 330 | 0.174 | 0.193 |
| Combined | collection5_k1_tropical_temperate | Transitional | Hothouse | 31 | 23 | 0.01 | 298 | 0.155 | 0.193 |
| Combined | collection5_k1_tropical_temperate | Warmhouse | Hothouse | 25 | 23 | 0.07 | 269 | 0.355 | 0.355 |
| Combined | collection5_k1_tropical_temperate_polar | Coldhouse | Warmhouse | 12 | 18 | -0.40 | 34 | 0.001 | 0.003 |
| Combined | collection5_k1_tropical_temperate_polar | Coldhouse | Hothouse | 12 | 18 | -0.34 | 35 | 0.001 | 0.003 |
| Combined | collection5_k1_tropical_temperate_polar | Coolhouse | Warmhouse | 21 | 18 | -0.20 | 79 | 0.001 | 0.003 |
| Combined | collection5_k1_tropical_temperate_polar | Coolhouse | Hothouse | 21 | 18 | -0.14 | 82 | 0.001 | 0.003 |
| Combined | collection5_k1_tropical_temperate_polar | Coldhouse | Transitional | 12 | 22 | -0.27 | 63 | 0.007 | 0.014 |
| Combined | collection5_k1_tropical_temperate_polar | Coolhouse | Transitional | 21 | 22 | -0.07 | 150 | 0.025 | 0.042 |
| Combined | collection5_k1_tropical_temperate_polar | Transitional | Warmhouse | 22 | 18 | -0.13 | 143 | 0.069 | 0.099 |
| Combined | collection5_k1_tropical_temperate_polar | Transitional | Hothouse | 22 | 18 | -0.06 | 150 | 0.098 | 0.123 |
| Combined | collection5_k1_tropical_temperate_polar | Coldhouse | Coolhouse | 12 | 21 | -0.20 | 101 | 0.180 | 0.200 |
| Combined | collection5_k1_tropical_temperate_polar | Warmhouse | Hothouse | 18 | 18 | 0.07 | 152 | 0.382 | 0.382 |
| Combined | collection5_k2_tropical_temperate | Coldhouse | Hothouse | 12 | 16 | -0.35 | 31 | 0.001 | 0.007 |
| Combined | collection5_k2_tropical_temperate | Coolhouse | Hothouse | 21 | 16 | -0.25 | 65 | 0.001 | 0.007 |
| Combined | collection5_k2_tropical_temperate | Coldhouse | Warmhouse | 12 | 17 | -0.25 | 49 | 0.010 | 0.033 |
| Combined | collection5_k2_tropical_temperate | Coolhouse | Warmhouse | 21 | 17 | -0.14 | 105 | 0.016 | 0.040 |
| Combined | collection5_k2_tropical_temperate | Coolhouse | Transitional | 21 | 20 | -0.25 | 134 | 0.024 | 0.049 |
| Combined | collection5_k2_tropical_temperate | Coldhouse | Transitional | 12 | 20 | -0.35 | 71 | 0.030 | 0.049 |
| Combined | collection5_k2_tropical_temperate | Transitional | Hothouse | 20 | 16 | 0.00 | 111 | 0.061 | 0.077 |
| Combined | collection5_k2_tropical_temperate | Warmhouse | Hothouse | 17 | 16 | -0.10 | 91 | 0.054 | 0.077 |
| Combined | collection5_k2_tropical_temperate | Coldhouse | Coolhouse | 12 | 21 | -0.10 | 114 | 0.333 | 0.371 |
| Combined | collection5_k2_tropical_temperate | Transitional | Warmhouse | 20 | 17 | 0.10 | 159 | 0.374 | 0.374 |
| Combined | collection5_k2_tropical_temperate_polar | Coldhouse | Transitional | 6 | 11 | -0.12 | 23 | 0.170 | 0.243 |
| Combined | collection5_k2_tropical_temperate_polar | Coldhouse | Warmhouse | 6 | 12 | -0.12 | 24 | 0.141 | 0.243 |
| Combined | collection5_k2_tropical_temperate_polar | Coldhouse | Hothouse | 6 | 7 | -0.21 | 9 | 0.050 | 0.243 |
| Combined | collection5_k2_tropical_temperate_polar | Coolhouse | Warmhouse | 14 | 12 | -0.03 | 59 | 0.104 | 0.243 |
| Combined | collection5_k2_tropical_temperate_polar | Coolhouse | Hothouse | 14 | 7 | -0.12 | 24 | 0.034 | 0.243 |
| Combined | collection5_k2_tropical_temperate_polar | Transitional | Hothouse | 11 | 7 | -0.09 | 27 | 0.160 | 0.243 |
| Combined | collection5_k2_tropical_temperate_polar | Warmhouse | Hothouse | 12 | 7 | -0.09 | 27 | 0.110 | 0.243 |
| Combined | collection5_k2_tropical_temperate_polar | Coolhouse | Transitional | 14 | 11 | -0.03 | 61 | 0.198 | 0.248 |
| Combined | collection5_k2_tropical_temperate_polar | Transitional | Warmhouse | 11 | 12 | 0.00 | 62 | 0.415 | 0.461 |
| Combined | collection5_k2_tropical_temperate_polar | Coldhouse | Coolhouse | 6 | 14 | -0.09 | 41 | 0.484 | 0.484 |
| Combined | collection5_k3_tropical_temperate | Coldhouse | Warmhouse | 6 | 14 | -0.35 | 11 | 0.006 | 0.030 |
| Combined | collection5_k3_tropical_temperate | Coldhouse | Hothouse | 6 | 11 | -0.38 | 7 | 0.005 | 0.030 |
| Combined | collection5_k3_tropical_temperate | Coolhouse | Hothouse | 7 | 11 | -0.12 | 13 | 0.012 | 0.039 |
| Combined | collection5_k3_tropical_temperate | Coldhouse | Transitional | 6 | 13 | -0.42 | 19 | 0.044 | 0.109 |
| Combined | collection5_k3_tropical_temperate | Coolhouse | Warmhouse | 7 | 14 | -0.09 | 28 | 0.063 | 0.126 |
| Combined | collection5_k3_tropical_temperate | Coolhouse | Transitional | 7 | 13 | -0.16 | 28 | 0.089 | 0.148 |
| Combined | collection5_k3_tropical_temperate | Warmhouse | Hothouse | 14 | 11 | -0.02 | 54 | 0.109 | 0.156 |
| Combined | collection5_k3_tropical_temperate | Coldhouse | Coolhouse | 6 | 7 | -0.26 | 15 | 0.216 | 0.240 |
| Combined | collection5_k3_tropical_temperate | Transitional | Hothouse | 13 | 11 | 0.04 | 56 | 0.192 | 0.240 |
| Combined | collection5_k3_tropical_temperate | Transitional | Warmhouse | 13 | 14 | 0.06 | 79 | 0.288 | 0.288 |
| Combined | collection5_k3_tropical_temperate_polar | Coldhouse | Coolhouse | 2 | 4 | -0.30 | 0 | 0.053 | 0.175 |
| Combined | collection5_k3_tropical_temperate_polar | Coldhouse | Warmhouse | 2 | 9 | -0.27 | 1 | 0.039 | 0.175 |
| Combined | collection5_k3_tropical_temperate_polar | Coldhouse | Hothouse | 2 | 5 | -0.37 | 0 | 0.041 | 0.175 |
| Combined | collection5_k3_tropical_temperate_polar | Coldhouse | Transitional | 2 | 5 | -0.21 | 1 | 0.088 | 0.182 |
| Combined | collection5_k3_tropical_temperate_polar | Warmhouse | Hothouse | 9 | 5 | -0.10 | 12 | 0.091 | 0.182 |
| Combined | collection5_k3_tropical_temperate_polar | Coolhouse | Hothouse | 4 | 5 | -0.08 | 5 | 0.135 | 0.225 |
| Combined | collection5_k3_tropical_temperate_polar | Transitional | Hothouse | 5 | 5 | -0.16 | 8 | 0.202 | 0.288 |
| Combined | collection5_k3_tropical_temperate_polar | Transitional | Warmhouse | 5 | 9 | -0.06 | 19 | 0.345 | 0.431 |
| Combined | collection5_k3_tropical_temperate_polar | Coolhouse | Warmhouse | 4 | 9 | 0.03 | 17 | 0.469 | 0.521 |
| Combined | collection5_k3_tropical_temperate_polar | Coolhouse | Transitional | 4 | 5 | 0.09 | 12 | 0.730 | 0.730 |
| Combined | collection10_k1_tropical_temperate | Coldhouse | Warmhouse | 16 | 20 | -0.42 | 61 | 0.001 | 0.003 |
| Combined | collection10_k1_tropical_temperate | Coldhouse | Hothouse | 16 | 20 | -0.50 | 54 | 0.000 | 0.003 |
| Combined | collection10_k1_tropical_temperate | Coolhouse | Hothouse | 25 | 20 | -0.36 | 109 | 0.001 | 0.003 |
| Combined | collection10_k1_tropical_temperate | Coolhouse | Warmhouse | 25 | 20 | -0.27 | 132 | 0.004 | 0.009 |
| Combined | collection10_k1_tropical_temperate | Coldhouse | Transitional | 16 | 24 | -0.32 | 116 | 0.019 | 0.037 |
| Combined | collection10_k1_tropical_temperate | Transitional | Hothouse | 24 | 20 | -0.18 | 164 | 0.038 | 0.063 |
| Combined | collection10_k1_tropical_temperate | Coolhouse | Transitional | 25 | 24 | -0.18 | 216 | 0.047 | 0.068 |
| Combined | collection10_k1_tropical_temperate | Transitional | Warmhouse | 24 | 20 | -0.10 | 192 | 0.131 | 0.164 |
| Combined | collection10_k1_tropical_temperate | Warmhouse | Hothouse | 20 | 20 | -0.09 | 168 | 0.197 | 0.219 |
| Combined | collection10_k1_tropical_temperate | Coldhouse | Coolhouse | 16 | 25 | -0.14 | 178 | 0.283 | 0.283 |
| Combined | collection10_k1_tropical_temperate_polar | Coldhouse | Warmhouse | 10 | 13 | -0.26 | 35 | 0.034 | 0.085 |
| Combined | collection10_k1_tropical_temperate_polar | Coldhouse | Hothouse | 10 | 8 | -0.32 | 20 | 0.042 | 0.085 |
| Combined | collection10_k1_tropical_temperate_polar | Coolhouse | Warmhouse | 14 | 13 | -0.18 | 55 | 0.042 | 0.085 |
| Combined | collection10_k1_tropical_temperate_polar | Coolhouse | Hothouse | 14 | 8 | -0.23 | 27 | 0.026 | 0.085 |
| Combined | collection10_k1_tropical_temperate_polar | Transitional | Hothouse | 13 | 8 | -0.30 | 23 | 0.020 | 0.085 |
| Combined | collection10_k1_tropical_temperate_polar | Transitional | Warmhouse | 13 | 13 | -0.25 | 53 | 0.056 | 0.093 |
| Combined | collection10_k1_tropical_temperate_polar | Warmhouse | Hothouse | 13 | 8 | -0.05 | 40 | 0.202 | 0.289 |
| Combined | collection10_k1_tropical_temperate_polar | Coldhouse | Coolhouse | 10 | 14 | -0.09 | 62 | 0.330 | 0.358 |
| Combined | collection10_k1_tropical_temperate_polar | Coldhouse | Transitional | 10 | 13 | -0.01 | 56 | 0.299 | 0.358 |
| Combined | collection10_k1_tropical_temperate_polar | Coolhouse | Transitional | 14 | 13 | 0.07 | 83 | 0.358 | 0.358 |
| Combined | collection10_k2_tropical_temperate | Coolhouse | Hothouse | 12 | 11 | -0.62 | 19 | 0.002 | 0.021 |
| Combined | collection10_k2_tropical_temperate | Coolhouse | Warmhouse | 12 | 15 | -0.34 | 42 | 0.010 | 0.051 |
| Combined | collection10_k2_tropical_temperate | Coldhouse | Hothouse | 9 | 11 | -0.48 | 21 | 0.017 | 0.056 |
| Combined | collection10_k2_tropical_temperate | Coolhouse | Transitional | 12 | 14 | -0.31 | 50 | 0.042 | 0.106 |
| Combined | collection10_k2_tropical_temperate | Coldhouse | Warmhouse | 9 | 15 | -0.20 | 42 | 0.068 | 0.136 |
| Combined | collection10_k2_tropical_temperate | Transitional | Hothouse | 14 | 11 | -0.31 | 54 | 0.109 | 0.156 |
| Combined | collection10_k2_tropical_temperate | Warmhouse | Hothouse | 15 | 11 | -0.28 | 57 | 0.097 | 0.156 |
| Combined | collection10_k2_tropical_temperate | Coldhouse | Transitional | 9 | 14 | -0.17 | 47 | 0.164 | 0.206 |
| Combined | collection10_k2_tropical_temperate | Transitional | Warmhouse | 14 | 15 | -0.02 | 97 | 0.372 | 0.413 |
| Combined | collection10_k2_tropical_temperate | Coldhouse | Coolhouse | 9 | 12 | 0.14 | 66 | 0.813 | 0.813 |
| Combined | collection10_k2_tropical_temperate_polar | Coldhouse | Warmhouse | 5 | 9 | -0.13 | 16 | 0.212 | 0.397 |
| Combined | collection10_k2_tropical_temperate_polar | Coldhouse | Hothouse | 5 | 3 | -0.48 | 3 | 0.117 | 0.397 |
| Combined | collection10_k2_tropical_temperate_polar | Coolhouse | Warmhouse | 5 | 9 | -0.03 | 17 | 0.252 | 0.397 |
| Combined | collection10_k2_tropical_temperate_polar | Coolhouse | Hothouse | 5 | 3 | -0.38 | 4 | 0.186 | 0.397 |
| Combined | collection10_k2_tropical_temperate_polar | Transitional | Warmhouse | 2 | 9 | -0.11 | 6 | 0.278 | 0.397 |
| Combined | collection10_k2_tropical_temperate_polar | Transitional | Hothouse | 2 | 3 | -0.46 | 1 | 0.193 | 0.397 |
| Combined | collection10_k2_tropical_temperate_polar | Warmhouse | Hothouse | 9 | 3 | -0.35 | 7 | 0.134 | 0.397 |
| Combined | collection10_k2_tropical_temperate_polar | Coldhouse | Coolhouse | 5 | 5 | -0.10 | 13 | 0.583 | 0.647 |
| Combined | collection10_k2_tropical_temperate_polar | Coolhouse | Transitional | 5 | 2 | 0.07 | 5 | 0.577 | 0.647 |
| Combined | collection10_k2_tropical_temperate_polar | Coldhouse | Transitional | 5 | 2 | -0.03 | 6 | 0.719 | 0.719 |
| Combined | collection10_k3_tropical_temperate | Coldhouse | Transitional | 4 | 7 | -0.27 | 6 | 0.078 | 0.353 |
| Combined | collection10_k3_tropical_temperate | Coldhouse | Warmhouse | 4 | 9 | -0.26 | 10 | 0.124 | 0.353 |
| Combined | collection10_k3_tropical_temperate | Coldhouse | Hothouse | 4 | 7 | -0.41 | 7 | 0.110 | 0.353 |
| Combined | collection10_k3_tropical_temperate | Coolhouse | Transitional | 6 | 7 | -0.10 | 13 | 0.142 | 0.353 |
| Combined | collection10_k3_tropical_temperate | Coolhouse | Hothouse | 6 | 7 | -0.24 | 14 | 0.177 | 0.353 |
| Combined | collection10_k3_tropical_temperate | Coolhouse | Warmhouse | 6 | 9 | -0.09 | 20 | 0.222 | 0.370 |
| Combined | collection10_k3_tropical_temperate | Coldhouse | Coolhouse | 4 | 6 | -0.17 | 9 | 0.297 | 0.424 |
| Combined | collection10_k3_tropical_temperate | Warmhouse | Hothouse | 9 | 7 | -0.15 | 28 | 0.375 | 0.469 |
| Combined | collection10_k3_tropical_temperate | Transitional | Hothouse | 7 | 7 | -0.14 | 24 | 0.500 | 0.556 |
| Combined | collection10_k3_tropical_temperate | Transitional | Warmhouse | 7 | 9 | 0.01 | 33 | 0.584 | 0.584 |
| Combined | collection10_k3_tropical_temperate_polar | Coolhouse | Hothouse | 3 | 2 | -0.56 | 0 | 0.074 | 0.112 |
| Combined | collection10_k3_tropical_temperate_polar | Warmhouse | Hothouse | 4 | 2 | -0.67 | 0 | 0.053 | 0.112 |
| Combined | collection10_k3_tropical_temperate_polar | Coolhouse | Warmhouse | 3 | 4 | 0.11 | 7 | 0.702 | 0.702 |
| Combined | collection10_k3_tropical_temperate_polar | Coldhouse | Coolhouse | 1 | 3 | -0.12 |  |  |  |
| Combined | collection10_k3_tropical_temperate_polar | Coldhouse | Transitional | 1 | 1 | -0.20 |  |  |  |
| Combined | collection10_k3_tropical_temperate_polar | Coldhouse | Warmhouse | 1 | 4 | -0.01 |  |  |  |
| Combined | collection10_k3_tropical_temperate_polar | Coldhouse | Hothouse | 1 | 2 | -0.67 |  |  |  |
| Combined | collection10_k3_tropical_temperate_polar | Coolhouse | Transitional | 3 | 1 | -0.09 |  |  |  |
| Combined | collection10_k3_tropical_temperate_polar | Transitional | Warmhouse | 1 | 4 | 0.20 |  |  |  |
| Combined | collection10_k3_tropical_temperate_polar | Transitional | Hothouse | 1 | 2 | -0.47 |  |  |  |
| Northern | occurrence5_k1_tropical_temperate | Coldhouse | Transitional | 12 | 17 | -0.38 | 37 | 0.002 | 0.008 |
| Northern | occurrence5_k1_tropical_temperate | Coldhouse | Warmhouse | 12 | 15 | -0.40 | 26 | 0.001 | 0.008 |
| Northern | occurrence5_k1_tropical_temperate | Coldhouse | Hothouse | 12 | 11 | -0.27 | 21 | 0.003 | 0.008 |
| Northern | occurrence5_k1_tropical_temperate | Coolhouse | Warmhouse | 15 | 15 | -0.23 | 45 | 0.003 | 0.008 |
| Northern | occurrence5_k1_tropical_temperate | Coolhouse | Transitional | 15 | 17 | -0.21 | 65 | 0.010 | 0.019 |
| Northern | occurrence5_k1_tropical_temperate | Coolhouse | Hothouse | 15 | 11 | -0.10 | 48 | 0.039 | 0.065 |
| Northern | occurrence5_k1_tropical_temperate | Coldhouse | Coolhouse | 12 | 15 | -0.18 | 69 | 0.159 | 0.227 |
| Northern | occurrence5_k1_tropical_temperate | Transitional | Warmhouse | 17 | 15 | -0.02 | 113 | 0.299 | 0.373 |
| Northern | occurrence5_k1_tropical_temperate | Transitional | Hothouse | 17 | 11 | 0.11 | 111 | 0.801 | 0.862 |
| Northern | occurrence5_k1_tropical_temperate | Warmhouse | Hothouse | 15 | 11 | 0.13 | 103 | 0.862 | 0.862 |
| Northern | occurrence5_k1_tropical_temperate_polar | Coldhouse | Transitional | 7 | 13 | -0.37 | 8 | 0.002 | 0.008 |
| Northern | occurrence5_k1_tropical_temperate_polar | Coldhouse | Warmhouse | 7 | 12 | -0.35 | 6 | 0.001 | 0.008 |
| Northern | occurrence5_k1_tropical_temperate_polar | Coldhouse | Hothouse | 7 | 7 | -0.22 | 2 | 0.002 | 0.008 |
| Northern | occurrence5_k1_tropical_temperate_polar | Coolhouse | Warmhouse | 12 | 12 | -0.14 | 36 | 0.020 | 0.051 |
| Northern | occurrence5_k1_tropical_temperate_polar | Coolhouse | Transitional | 12 | 13 | -0.16 | 43 | 0.030 | 0.061 |
| Northern | occurrence5_k1_tropical_temperate_polar | Coldhouse | Coolhouse | 7 | 12 | -0.21 | 25 | 0.082 | 0.136 |
| Northern | occurrence5_k1_tropical_temperate_polar | Coolhouse | Hothouse | 12 | 7 | -0.01 | 35 | 0.291 | 0.416 |
| Northern | occurrence5_k1_tropical_temperate_polar | Transitional | Warmhouse | 13 | 12 | 0.02 | 76 | 0.467 | 0.584 |
| Northern | occurrence5_k1_tropical_temperate_polar | Transitional | Hothouse | 13 | 7 | 0.14 | 63 | 0.923 | 0.930 |
| Northern | occurrence5_k1_tropical_temperate_polar | Warmhouse | Hothouse | 12 | 7 | 0.12 | 59 | 0.930 | 0.930 |
| Northern | occurrence5_k2_tropical_temperate | Coldhouse | Transitional | 8 | 15 | -0.30 | 24 | 0.011 | 0.033 |
| Northern | occurrence5_k2_tropical_temperate | Coldhouse | Warmhouse | 8 | 12 | -0.31 | 16 | 0.008 | 0.033 |
| Northern | occurrence5_k2_tropical_temperate | Coldhouse | Hothouse | 8 | 8 | -0.24 | 9 | 0.009 | 0.033 |
| Northern | occurrence5_k2_tropical_temperate | Coolhouse | Warmhouse | 12 | 12 | -0.21 | 33 | 0.013 | 0.033 |
| Northern | occurrence5_k2_tropical_temperate | Coolhouse | Transitional | 12 | 15 | -0.20 | 52 | 0.034 | 0.067 |
| Northern | occurrence5_k2_tropical_temperate | Coolhouse | Hothouse | 12 | 8 | -0.14 | 31 | 0.102 | 0.169 |
| Northern | occurrence5_k2_tropical_temperate | Coldhouse | Coolhouse | 8 | 12 | -0.10 | 38 | 0.232 | 0.331 |
| Northern | occurrence5_k2_tropical_temperate | Transitional | Warmhouse | 15 | 12 | -0.01 | 78 | 0.287 | 0.359 |
| Northern | occurrence5_k2_tropical_temperate | Transitional | Hothouse | 15 | 8 | 0.06 | 68 | 0.708 | 0.787 |
| Northern | occurrence5_k2_tropical_temperate | Warmhouse | Hothouse | 12 | 8 | 0.07 | 59 | 0.813 | 0.813 |
| Northern | occurrence5_k2_tropical_temperate_polar | Coldhouse | Transitional | 5 | 8 | -0.22 | 5 | 0.017 | 0.056 |
| Northern | occurrence5_k2_tropical_temperate_polar | Coldhouse | Warmhouse | 5 | 10 | -0.26 | 6 | 0.012 | 0.056 |
| Northern | occurrence5_k2_tropical_temperate_polar | Coldhouse | Hothouse | 5 | 6 | -0.16 | 2 | 0.011 | 0.056 |
| Northern | occurrence5_k2_tropical_temperate_polar | Coldhouse | Coolhouse | 5 | 8 | -0.20 | 7 | 0.034 | 0.084 |
| Northern | occurrence5_k2_tropical_temperate_polar | Coolhouse | Warmhouse | 8 | 10 | -0.06 | 31 | 0.225 | 0.450 |
| Northern | occurrence5_k2_tropical_temperate_polar | Transitional | Warmhouse | 8 | 10 | -0.04 | 36 | 0.378 | 0.630 |
| Northern | occurrence5_k2_tropical_temperate_polar | Coolhouse | Transitional | 8 | 8 | -0.02 | 31 | 0.479 | 0.684 |
| Northern | occurrence5_k2_tropical_temperate_polar | Coolhouse | Hothouse | 8 | 6 | 0.05 | 28 | 0.719 | 0.873 |
| Northern | occurrence5_k2_tropical_temperate_polar | Transitional | Hothouse | 8 | 6 | 0.06 | 31 | 0.834 | 0.873 |
| Northern | occurrence5_k2_tropical_temperate_polar | Warmhouse | Hothouse | 10 | 6 | 0.11 | 40 | 0.873 | 0.873 |
| Northern | occurrence5_k3_tropical_temperate | Coolhouse | Transitional | 7 | 12 | -0.32 | 13 | 0.008 | 0.046 |
| Northern | occurrence5_k3_tropical_temperate | Coolhouse | Warmhouse | 7 | 11 | -0.29 | 12 | 0.009 | 0.046 |
| Northern | occurrence5_k3_tropical_temperate | Coldhouse | Transitional | 7 | 12 | -0.30 | 18 | 0.024 | 0.054 |
| Northern | occurrence5_k3_tropical_temperate | Coldhouse | Warmhouse | 7 | 11 | -0.27 | 15 | 0.019 | 0.054 |
| Northern | occurrence5_k3_tropical_temperate | Coldhouse | Hothouse | 7 | 6 | -0.21 | 7 | 0.027 | 0.054 |
| Northern | occurrence5_k3_tropical_temperate | Coolhouse | Hothouse | 7 | 6 | -0.23 | 9 | 0.050 | 0.084 |
| Northern | occurrence5_k3_tropical_temperate | Coldhouse | Coolhouse | 7 | 7 | 0.01 | 26 | 0.601 | 0.751 |
| Northern | occurrence5_k3_tropical_temperate | Transitional | Warmhouse | 12 | 11 | 0.03 | 69 | 0.585 | 0.751 |
| Northern | occurrence5_k3_tropical_temperate | Transitional | Hothouse | 12 | 6 | 0.09 | 44 | 0.787 | 0.787 |
| Northern | occurrence5_k3_tropical_temperate | Warmhouse | Hothouse | 11 | 6 | 0.06 | 38 | 0.710 | 0.787 |
| Northern | occurrence5_k3_tropical_temperate_polar | Coldhouse | Transitional | 4 | 6 | -0.27 | 3 | 0.035 | 0.124 |
| Northern | occurrence5_k3_tropical_temperate_polar | Coldhouse | Warmhouse | 4 | 8 | -0.21 | 5 | 0.037 | 0.124 |
| Northern | occurrence5_k3_tropical_temperate_polar | Coldhouse | Hothouse | 4 | 3 | -0.25 | 0 | 0.026 | 0.124 |
| Northern | occurrence5_k3_tropical_temperate_polar | Coolhouse | Transitional | 4 | 6 | -0.17 | 6 | 0.120 | 0.301 |
| Northern | occurrence5_k3_tropical_temperate_polar | Coolhouse | Warmhouse | 4 | 8 | -0.11 | 10 | 0.175 | 0.350 |
| Northern | occurrence5_k3_tropical_temperate_polar | Coldhouse | Coolhouse | 4 | 4 | -0.10 | 5 | 0.235 | 0.392 |
| Northern | occurrence5_k3_tropical_temperate_polar | Coolhouse | Hothouse | 4 | 3 | -0.15 | 4 | 0.298 | 0.426 |
| Northern | occurrence5_k3_tropical_temperate_polar | Warmhouse | Hothouse | 8 | 3 | -0.04 | 11 | 0.459 | 0.574 |
| Northern | occurrence5_k3_tropical_temperate_polar | Transitional | Warmhouse | 6 | 8 | 0.06 | 30 | 0.799 | 0.799 |
| Northern | occurrence5_k3_tropical_temperate_polar | Transitional | Hothouse | 6 | 3 | 0.02 | 11 | 0.741 | 0.799 |
| Northern | occurrence10_k1_tropical_temperate | Coldhouse | Warmhouse | 11 | 15 | -0.79 | 9 | 0.000 | 0.001 |
| Northern | occurrence10_k1_tropical_temperate | Coldhouse | Transitional | 11 | 17 | -0.67 | 24 | 0.001 | 0.003 |
| Northern | occurrence10_k1_tropical_temperate | Coldhouse | Hothouse | 11 | 11 | -0.53 | 19 | 0.004 | 0.009 |
| Northern | occurrence10_k1_tropical_temperate | Coolhouse | Warmhouse | 15 | 15 | -0.35 | 46 | 0.003 | 0.009 |
| Northern | occurrence10_k1_tropical_temperate | Coldhouse | Coolhouse | 11 | 15 | -0.44 | 46 | 0.031 | 0.058 |
| Northern | occurrence10_k1_tropical_temperate | Coolhouse | Transitional | 15 | 17 | -0.23 | 79 | 0.035 | 0.058 |
| Northern | occurrence10_k1_tropical_temperate | Transitional | Warmhouse | 17 | 15 | -0.12 | 90 | 0.081 | 0.116 |
| Northern | occurrence10_k1_tropical_temperate | Coolhouse | Hothouse | 15 | 11 | -0.09 | 58 | 0.106 | 0.133 |
| Northern | occurrence10_k1_tropical_temperate | Transitional | Hothouse | 17 | 11 | 0.14 | 111 | 0.801 | 0.890 |
| Northern | occurrence10_k1_tropical_temperate | Warmhouse | Hothouse | 15 | 11 | 0.26 | 116 | 0.961 | 0.961 |
| Northern | occurrence10_k1_tropical_temperate_polar | Coldhouse | Warmhouse | 5 | 12 | -0.36 | 3 | 0.003 | 0.026 |
| Northern | occurrence10_k1_tropical_temperate_polar | Coldhouse | Transitional | 5 | 13 | -0.28 | 7 | 0.007 | 0.034 |
| Northern | occurrence10_k1_tropical_temperate_polar | Coldhouse | Coolhouse | 5 | 9 | -0.20 | 6 | 0.016 | 0.055 |
| Northern | occurrence10_k1_tropical_temperate_polar | Coldhouse | Hothouse | 5 | 5 | -0.13 | 5 | 0.072 | 0.180 |
| Northern | occurrence10_k1_tropical_temperate_polar | Coolhouse | Warmhouse | 9 | 12 | -0.16 | 37 | 0.120 | 0.241 |
| Northern | occurrence10_k1_tropical_temperate_polar | Transitional | Warmhouse | 13 | 12 | -0.08 | 58 | 0.144 | 0.241 |
| Northern | occurrence10_k1_tropical_temperate_polar | Coolhouse | Transitional | 9 | 13 | -0.08 | 49 | 0.274 | 0.391 |
| Northern | occurrence10_k1_tropical_temperate_polar | Coolhouse | Hothouse | 9 | 5 | 0.07 | 27 | 0.748 | 0.934 |
| Northern | occurrence10_k1_tropical_temperate_polar | Transitional | Hothouse | 13 | 5 | 0.15 | 48 | 0.943 | 0.959 |
| Northern | occurrence10_k1_tropical_temperate_polar | Warmhouse | Hothouse | 12 | 5 | 0.23 | 46 | 0.959 | 0.959 |
| Northern | occurrence10_k2_tropical_temperate | Coldhouse | Warmhouse | 8 | 11 | -0.52 | 8 | 0.002 | 0.017 |
| Northern | occurrence10_k2_tropical_temperate | Coldhouse | Transitional | 8 | 14 | -0.41 | 19 | 0.006 | 0.032 |
| Northern | occurrence10_k2_tropical_temperate | Coolhouse | Warmhouse | 10 | 11 | -0.36 | 24 | 0.016 | 0.053 |
| Northern | occurrence10_k2_tropical_temperate | Coldhouse | Hothouse | 8 | 8 | -0.32 | 14 | 0.033 | 0.083 |
| Northern | occurrence10_k2_tropical_temperate | Coolhouse | Transitional | 10 | 14 | -0.24 | 42 | 0.054 | 0.107 |
| Northern | occurrence10_k2_tropical_temperate | Coolhouse | Hothouse | 10 | 8 | -0.15 | 26 | 0.115 | 0.171 |
| Northern | occurrence10_k2_tropical_temperate | Transitional | Warmhouse | 14 | 11 | -0.12 | 55 | 0.120 | 0.171 |
| Northern | occurrence10_k2_tropical_temperate | Coldhouse | Coolhouse | 8 | 10 | -0.16 | 30 | 0.199 | 0.249 |
| Northern | occurrence10_k2_tropical_temperate | Transitional | Hothouse | 14 | 8 | 0.09 | 65 | 0.742 | 0.824 |
| Northern | occurrence10_k2_tropical_temperate | Warmhouse | Hothouse | 11 | 8 | 0.21 | 57 | 0.868 | 0.868 |
| Northern | occurrence10_k2_tropical_temperate_polar | Coldhouse | Transitional | 4 | 8 | -0.22 | 4 | 0.025 | 0.127 |
| Northern | occurrence10_k2_tropical_temperate_polar | Coldhouse | Warmhouse | 4 | 7 | -0.14 | 3 | 0.024 | 0.127 |
| Northern | occurrence10_k2_tropical_temperate_polar | Coldhouse | Coolhouse | 4 | 5 | -0.22 | 3 | 0.056 | 0.140 |
| Northern | occurrence10_k2_tropical_temperate_polar | Coldhouse | Hothouse | 4 | 4 | -0.17 | 2 | 0.056 | 0.140 |
| Northern | occurrence10_k2_tropical_temperate_polar | Coolhouse | Transitional | 5 | 8 | 0.00 | 20 | 0.529 | 0.768 |
| Northern | occurrence10_k2_tropical_temperate_polar | Transitional | Warmhouse | 8 | 7 | 0.08 | 28 | 0.523 | 0.768 |
| Northern | occurrence10_k2_tropical_temperate_polar | Warmhouse | Hothouse | 7 | 4 | -0.04 | 14 | 0.538 | 0.768 |
| Northern | occurrence10_k2_tropical_temperate_polar | Coolhouse | Warmhouse | 5 | 7 | 0.08 | 19 | 0.627 | 0.778 |
| Northern | occurrence10_k2_tropical_temperate_polar | Coolhouse | Hothouse | 5 | 4 | 0.05 | 12 | 0.730 | 0.778 |
| Northern | occurrence10_k2_tropical_temperate_polar | Transitional | Hothouse | 8 | 4 | 0.05 | 20 | 0.778 | 0.778 |
| Northern | occurrence10_k3_tropical_temperate | Coldhouse | Transitional | 7 | 11 | -0.32 | 11 | 0.007 | 0.036 |
| Northern | occurrence10_k3_tropical_temperate | Coldhouse | Warmhouse | 7 | 9 | -0.40 | 7 | 0.006 | 0.036 |
| Northern | occurrence10_k3_tropical_temperate | Coolhouse | Transitional | 7 | 11 | -0.40 | 13 | 0.012 | 0.039 |
| Northern | occurrence10_k3_tropical_temperate | Coolhouse | Warmhouse | 7 | 9 | -0.47 | 11 | 0.017 | 0.043 |
| Northern | occurrence10_k3_tropical_temperate | Coldhouse | Hothouse | 7 | 6 | -0.19 | 10 | 0.067 | 0.134 |
| Northern | occurrence10_k3_tropical_temperate | Coolhouse | Hothouse | 7 | 6 | -0.26 | 12 | 0.112 | 0.187 |
| Northern | occurrence10_k3_tropical_temperate | Transitional | Warmhouse | 11 | 9 | -0.07 | 41 | 0.272 | 0.388 |
| Northern | occurrence10_k3_tropical_temperate | Coldhouse | Coolhouse | 7 | 7 | 0.07 | 22 | 0.399 | 0.499 |
| Northern | occurrence10_k3_tropical_temperate | Transitional | Hothouse | 11 | 6 | 0.13 | 43 | 0.854 | 0.854 |
| Northern | occurrence10_k3_tropical_temperate | Warmhouse | Hothouse | 9 | 6 | 0.21 | 33 | 0.778 | 0.854 |
| Northern | occurrence10_k3_tropical_temperate_polar | Coldhouse | Transitional | 4 | 5 | -0.26 | 3 | 0.056 | 0.278 |
| Northern | occurrence10_k3_tropical_temperate_polar | Coldhouse | Warmhouse | 4 | 5 | -0.11 | 3 | 0.056 | 0.278 |
| Northern | occurrence10_k3_tropical_temperate_polar | Coldhouse | Hothouse | 4 | 3 | -0.23 | 2 | 0.108 | 0.360 |
| Northern | occurrence10_k3_tropical_temperate_polar | Coolhouse | Transitional | 2 | 5 | -0.24 | 3 | 0.281 | 0.561 |
| Northern | occurrence10_k3_tropical_temperate_polar | Coolhouse | Warmhouse | 2 | 5 | -0.09 | 3 | 0.281 | 0.561 |
| Northern | occurrence10_k3_tropical_temperate_polar | Coldhouse | Coolhouse | 4 | 2 | -0.02 | 3 | 0.408 | 0.681 |
| Northern | occurrence10_k3_tropical_temperate_polar | Coolhouse | Hothouse | 2 | 3 | -0.21 | 3 | 0.614 | 0.686 |
| Northern | occurrence10_k3_tropical_temperate_polar | Transitional | Warmhouse | 5 | 5 | 0.15 | 12 | 0.500 | 0.686 |
| Northern | occurrence10_k3_tropical_temperate_polar | Warmhouse | Hothouse | 5 | 3 | -0.12 | 8 | 0.617 | 0.686 |
| Northern | occurrence10_k3_tropical_temperate_polar | Transitional | Hothouse | 5 | 3 | 0.03 | 10 | 0.814 | 0.814 |
| Northern | collection5_k1_tropical_temperate | Coldhouse | Warmhouse | 11 | 14 | -0.63 | 10 | 0.000 | 0.001 |
| Northern | collection5_k1_tropical_temperate | Coldhouse | Transitional | 11 | 16 | -0.63 | 25 | 0.001 | 0.005 |
| Northern | collection5_k1_tropical_temperate | Coolhouse | Warmhouse | 14 | 14 | -0.22 | 42 | 0.005 | 0.018 |
| Northern | collection5_k1_tropical_temperate | Coldhouse | Hothouse | 11 | 10 | -0.53 | 21 | 0.009 | 0.023 |
| Northern | collection5_k1_tropical_temperate | Coolhouse | Transitional | 14 | 16 | -0.23 | 57 | 0.012 | 0.023 |
| Northern | collection5_k1_tropical_temperate | Coldhouse | Coolhouse | 11 | 14 | -0.41 | 52 | 0.090 | 0.134 |
| Northern | collection5_k1_tropical_temperate | Coolhouse | Hothouse | 14 | 10 | -0.12 | 47 | 0.094 | 0.134 |
| Northern | collection5_k1_tropical_temperate | Transitional | Warmhouse | 16 | 14 | 0.01 | 92 | 0.209 | 0.261 |
| Northern | collection5_k1_tropical_temperate | Transitional | Hothouse | 16 | 10 | 0.10 | 97 | 0.822 | 0.822 |
| Northern | collection5_k1_tropical_temperate | Warmhouse | Hothouse | 14 | 10 | 0.10 | 81 | 0.750 | 0.822 |
| Northern | collection5_k1_tropical_temperate_polar | Coldhouse | Warmhouse | 7 | 10 | -0.34 | 3 | 0.001 | 0.011 |
| Northern | collection5_k1_tropical_temperate_polar | Coldhouse | Transitional | 7 | 11 | -0.45 | 9 | 0.004 | 0.014 |
| Northern | collection5_k1_tropical_temperate_polar | Coldhouse | Hothouse | 7 | 7 | -0.36 | 3 | 0.004 | 0.014 |
| Northern | collection5_k1_tropical_temperate_polar | Coldhouse | Coolhouse | 7 | 11 | -0.31 | 17 | 0.029 | 0.071 |
| Northern | collection5_k1_tropical_temperate_polar | Coolhouse | Warmhouse | 11 | 10 | -0.03 | 35 | 0.085 | 0.170 |
| Northern | collection5_k1_tropical_temperate_polar | Coolhouse | Transitional | 11 | 11 | -0.14 | 42 | 0.119 | 0.198 |
| Northern | collection5_k1_tropical_temperate_polar | Coolhouse | Hothouse | 11 | 7 | -0.05 | 30 | 0.234 | 0.335 |
| Northern | collection5_k1_tropical_temperate_polar | Transitional | Warmhouse | 11 | 10 | 0.11 | 48 | 0.324 | 0.404 |
| Northern | collection5_k1_tropical_temperate_polar | Warmhouse | Hothouse | 10 | 7 | -0.02 | 36 | 0.558 | 0.620 |
| Northern | collection5_k1_tropical_temperate_polar | Transitional | Hothouse | 11 | 7 | 0.08 | 48 | 0.817 | 0.817 |
| Northern | collection5_k2_tropical_temperate | Coldhouse | Warmhouse | 7 | 10 | -0.34 | 8 | 0.005 | 0.049 |
| Northern | collection5_k2_tropical_temperate | Coldhouse | Hothouse | 7 | 7 | -0.37 | 6 | 0.011 | 0.054 |
| Northern | collection5_k2_tropical_temperate | Coldhouse | Transitional | 7 | 14 | -0.47 | 20 | 0.017 | 0.056 |
| Northern | collection5_k2_tropical_temperate | Coolhouse | Transitional | 10 | 14 | -0.21 | 40 | 0.042 | 0.104 |
| Northern | collection5_k2_tropical_temperate | Coolhouse | Warmhouse | 10 | 10 | -0.08 | 28 | 0.052 | 0.104 |
| Northern | collection5_k2_tropical_temperate | Coolhouse | Hothouse | 10 | 7 | -0.11 | 20 | 0.079 | 0.131 |
| Northern | collection5_k2_tropical_temperate | Coldhouse | Coolhouse | 7 | 10 | -0.26 | 28 | 0.263 | 0.329 |
| Northern | collection5_k2_tropical_temperate | Warmhouse | Hothouse | 10 | 7 | -0.03 | 27 | 0.232 | 0.329 |
| Northern | collection5_k2_tropical_temperate | Transitional | Warmhouse | 14 | 10 | 0.13 | 61 | 0.309 | 0.344 |
| Northern | collection5_k2_tropical_temperate | Transitional | Hothouse | 14 | 7 | 0.10 | 50 | 0.545 | 0.545 |
| Northern | collection5_k2_tropical_temperate_polar | Coldhouse | Coolhouse | 3 | 7 | -0.35 | 2 | 0.034 | 0.101 |
| Northern | collection5_k2_tropical_temperate_polar | Coldhouse | Transitional | 3 | 7 | -0.30 | 2 | 0.034 | 0.101 |
| Northern | collection5_k2_tropical_temperate_polar | Coldhouse | Warmhouse | 3 | 8 | -0.32 | 1 | 0.016 | 0.101 |
| Northern | collection5_k2_tropical_temperate_polar | Coldhouse | Hothouse | 3 | 3 | -0.38 | 0 | 0.040 | 0.101 |
| Northern | collection5_k2_tropical_temperate_polar | Warmhouse | Hothouse | 8 | 3 | -0.06 | 9 | 0.305 | 0.610 |
| Northern | collection5_k2_tropical_temperate_polar | Coolhouse | Transitional | 7 | 7 | 0.04 | 27 | 0.649 | 0.649 |
| Northern | collection5_k2_tropical_temperate_polar | Coolhouse | Warmhouse | 7 | 8 | 0.03 | 28 | 0.523 | 0.649 |
| Northern | collection5_k2_tropical_temperate_polar | Coolhouse | Hothouse | 7 | 3 | -0.03 | 11 | 0.590 | 0.649 |
| Northern | collection5_k2_tropical_temperate_polar | Transitional | Warmhouse | 7 | 8 | -0.01 | 26 | 0.431 | 0.649 |
| Northern | collection5_k2_tropical_temperate_polar | Transitional | Hothouse | 7 | 3 | -0.07 | 11 | 0.590 | 0.649 |
| Northern | collection5_k3_tropical_temperate | Coldhouse | Transitional | 6 | 10 | -0.47 | 11 | 0.022 | 0.076 |
| Northern | collection5_k3_tropical_temperate | Coldhouse | Warmhouse | 6 | 10 | -0.29 | 8 | 0.010 | 0.076 |
| Northern | collection5_k3_tropical_temperate | Coldhouse | Hothouse | 6 | 6 | -0.32 | 5 | 0.023 | 0.076 |
| Northern | collection5_k3_tropical_temperate | Coolhouse | Transitional | 4 | 10 | -0.42 | 7 | 0.039 | 0.077 |
| Northern | collection5_k3_tropical_temperate | Coolhouse | Warmhouse | 4 | 10 | -0.24 | 7 | 0.039 | 0.077 |
| Northern | collection5_k3_tropical_temperate | Coolhouse | Hothouse | 4 | 6 | -0.27 | 5 | 0.083 | 0.138 |
| Northern | collection5_k3_tropical_temperate | Warmhouse | Hothouse | 10 | 6 | -0.03 | 23 | 0.240 | 0.343 |
| Northern | collection5_k3_tropical_temperate | Coldhouse | Coolhouse | 6 | 4 | -0.05 | 12 | 0.542 | 0.603 |
| Northern | collection5_k3_tropical_temperate | Transitional | Warmhouse | 10 | 10 | 0.18 | 49 | 0.485 | 0.603 |
| Northern | collection5_k3_tropical_temperate | Transitional | Hothouse | 10 | 6 | 0.15 | 36 | 0.760 | 0.760 |
| Northern | collection5_k3_tropical_temperate_polar | Coldhouse | Coolhouse | 2 | 2 | -0.30 | 0 | 0.123 | 0.409 |
| Northern | collection5_k3_tropical_temperate_polar | Coldhouse | Warmhouse | 2 | 8 | -0.26 | 1 | 0.045 | 0.409 |
| Northern | collection5_k3_tropical_temperate_polar | Coldhouse | Hothouse | 2 | 2 | -0.26 | 0 | 0.123 | 0.409 |
| Northern | collection5_k3_tropical_temperate_polar | Coldhouse | Transitional | 2 | 3 | -0.17 | 1 | 0.193 | 0.483 |
| Northern | collection5_k3_tropical_temperate_polar | Coolhouse | Warmhouse | 2 | 8 | 0.04 | 7 | 0.448 | 0.640 |
| Northern | collection5_k3_tropical_temperate_polar | Transitional | Warmhouse | 3 | 8 | -0.10 | 10 | 0.380 | 0.640 |
| Northern | collection5_k3_tropical_temperate_polar | Warmhouse | Hothouse | 8 | 2 | 0.00 | 7 | 0.448 | 0.640 |
| Northern | collection5_k3_tropical_temperate_polar | Coolhouse | Hothouse | 2 | 2 | 0.04 | 2 | 0.651 | 0.723 |
| Northern | collection5_k3_tropical_temperate_polar | Transitional | Hothouse | 3 | 2 | -0.09 | 3 | 0.614 | 0.723 |
| Northern | collection5_k3_tropical_temperate_polar | Coolhouse | Transitional | 2 | 3 | 0.13 | 4 | 0.807 | 0.807 |
| Northern | collection10_k1_tropical_temperate | Coldhouse | Warmhouse | 9 | 13 | -0.76 | 10 | 0.001 | 0.007 |
| Northern | collection10_k1_tropical_temperate | Coldhouse | Transitional | 9 | 13 | -0.49 | 20 | 0.006 | 0.028 |
| Northern | collection10_k1_tropical_temperate | Coldhouse | Hothouse | 9 | 9 | -0.52 | 13 | 0.009 | 0.029 |
| Northern | collection10_k1_tropical_temperate | Coolhouse | Warmhouse | 12 | 13 | -0.32 | 40 | 0.021 | 0.052 |
| Northern | collection10_k1_tropical_temperate | Coldhouse | Coolhouse | 9 | 12 | -0.44 | 35 | 0.094 | 0.184 |
| Northern | collection10_k1_tropical_temperate | Coolhouse | Transitional | 12 | 13 | -0.05 | 55 | 0.111 | 0.184 |
| Northern | collection10_k1_tropical_temperate | Transitional | Warmhouse | 13 | 13 | -0.27 | 69 | 0.221 | 0.316 |
| Northern | collection10_k1_tropical_temperate | Coolhouse | Hothouse | 12 | 9 | -0.09 | 45 | 0.273 | 0.341 |
| Northern | collection10_k1_tropical_temperate | Transitional | Hothouse | 13 | 9 | -0.03 | 64 | 0.656 | 0.729 |
| Northern | collection10_k1_tropical_temperate | Warmhouse | Hothouse | 13 | 9 | 0.24 | 78 | 0.909 | 0.909 |
| Northern | collection10_k1_tropical_temperate_polar | Coldhouse | Warmhouse | 5 | 9 | -0.47 | 4 | 0.008 | 0.082 |
| Northern | collection10_k1_tropical_temperate_polar | Coldhouse | Coolhouse | 5 | 7 | -0.44 | 5 | 0.026 | 0.128 |
| Northern | collection10_k1_tropical_temperate_polar | Coldhouse | Transitional | 5 | 6 | -0.24 | 6 | 0.060 | 0.201 |
| Northern | collection10_k1_tropical_temperate_polar | Coldhouse | Hothouse | 5 | 3 | -0.30 | 3 | 0.117 | 0.263 |
| Northern | collection10_k1_tropical_temperate_polar | Transitional | Warmhouse | 6 | 9 | -0.23 | 17 | 0.131 | 0.263 |
| Northern | collection10_k1_tropical_temperate_polar | Coolhouse | Warmhouse | 7 | 9 | -0.03 | 27 | 0.336 | 0.560 |
| Northern | collection10_k1_tropical_temperate_polar | Transitional | Hothouse | 6 | 3 | -0.06 | 8 | 0.449 | 0.641 |
| Northern | collection10_k1_tropical_temperate_polar | Coolhouse | Transitional | 7 | 6 | 0.20 | 25 | 0.740 | 0.837 |
| Northern | collection10_k1_tropical_temperate_polar | Coolhouse | Hothouse | 7 | 3 | 0.14 | 13 | 0.753 | 0.837 |
| Northern | collection10_k1_tropical_temperate_polar | Warmhouse | Hothouse | 9 | 3 | 0.17 | 19 | 0.866 | 0.866 |
| Northern | collection10_k2_tropical_temperate | Coldhouse | Transitional | 6 | 10 | -0.49 | 14 | 0.046 | 0.128 |
| Northern | collection10_k2_tropical_temperate | Coldhouse | Warmhouse | 6 | 8 | -0.61 | 7 | 0.017 | 0.128 |
| Northern | collection10_k2_tropical_temperate | Coldhouse | Hothouse | 6 | 6 | -0.43 | 8 | 0.064 | 0.128 |
| Northern | collection10_k2_tropical_temperate | Coolhouse | Transitional | 7 | 10 | -0.57 | 18 | 0.054 | 0.128 |
| Northern | collection10_k2_tropical_temperate | Coolhouse | Warmhouse | 7 | 8 | -0.69 | 11 | 0.028 | 0.128 |
| Northern | collection10_k2_tropical_temperate | Coolhouse | Hothouse | 7 | 6 | -0.51 | 11 | 0.087 | 0.146 |
| Northern | collection10_k2_tropical_temperate | Transitional | Warmhouse | 10 | 8 | -0.12 | 34 | 0.313 | 0.446 |
| Northern | collection10_k2_tropical_temperate | Coldhouse | Coolhouse | 6 | 7 | 0.08 | 23 | 0.640 | 0.719 |
| Northern | collection10_k2_tropical_temperate | Transitional | Hothouse | 10 | 6 | 0.06 | 34 | 0.687 | 0.719 |
| Northern | collection10_k2_tropical_temperate | Warmhouse | Hothouse | 8 | 6 | 0.18 | 28 | 0.719 | 0.719 |
| Northern | collection10_k2_tropical_temperate_polar | Coldhouse | Coolhouse | 3 | 3 | -0.23 | 2 | 0.191 | 0.287 |
| Northern | collection10_k2_tropical_temperate_polar | Coldhouse | Warmhouse | 3 | 5 | -0.16 | 3 | 0.117 | 0.287 |
| Northern | collection10_k2_tropical_temperate_polar | Coolhouse | Warmhouse | 3 | 5 | 0.07 | 7 | 0.500 | 0.500 |
| Northern | collection10_k2_tropical_temperate_polar | Coldhouse | Transitional | 3 | 1 | 0.02 |  |  |  |
| Northern | collection10_k2_tropical_temperate_polar | Coldhouse | Hothouse | 3 | 1 | -0.09 |  |  |  |
| Northern | collection10_k2_tropical_temperate_polar | Coolhouse | Transitional | 3 | 1 | 0.25 |  |  |  |
| Northern | collection10_k2_tropical_temperate_polar | Coolhouse | Hothouse | 3 | 1 | 0.14 |  |  |  |
| Northern | collection10_k2_tropical_temperate_polar | Transitional | Warmhouse | 1 | 5 | -0.18 |  |  |  |
| Northern | collection10_k2_tropical_temperate_polar | Transitional | Hothouse | 1 | 1 | -0.11 |  |  |  |
| Northern | collection10_k2_tropical_temperate_polar | Warmhouse | Hothouse | 5 | 1 | 0.07 |  |  |  |
| Northern | collection10_k3_tropical_temperate | Coldhouse | Transitional | 4 | 5 | -0.48 | 4 | 0.089 | 0.583 |
| Northern | collection10_k3_tropical_temperate | Coolhouse | Transitional | 3 | 5 | -0.31 | 3 | 0.117 | 0.583 |
| Northern | collection10_k3_tropical_temperate | Coldhouse | Warmhouse | 4 | 5 | -0.20 | 6 | 0.196 | 0.652 |
| Northern | collection10_k3_tropical_temperate | Coolhouse | Warmhouse | 3 | 5 | -0.03 | 5 | 0.275 | 0.689 |
| Northern | collection10_k3_tropical_temperate | Coldhouse | Coolhouse | 4 | 3 | -0.16 | 5 | 0.430 | 0.728 |
| Northern | collection10_k3_tropical_temperate | Coldhouse | Hothouse | 4 | 4 | -0.09 | 7 | 0.443 | 0.728 |
| Northern | collection10_k3_tropical_temperate | Coolhouse | Hothouse | 3 | 4 | 0.08 | 6 | 0.570 | 0.728 |
| Northern | collection10_k3_tropical_temperate | Transitional | Warmhouse | 5 | 5 | 0.28 | 13 | 0.583 | 0.728 |
| Northern | collection10_k3_tropical_temperate | Warmhouse | Hothouse | 5 | 4 | 0.11 | 13 | 0.804 | 0.894 |
| Northern | collection10_k3_tropical_temperate | Transitional | Hothouse | 5 | 4 | 0.39 | 16 | 0.944 | 0.944 |
| Northern | collection10_k3_tropical_temperate_polar | Coldhouse | Coolhouse | 1 | 1 | -0.28 |  |  |  |
| Northern | collection10_k3_tropical_temperate_polar | Coldhouse | Transitional | 1 | 0 |  |  |  |  |
| Northern | collection10_k3_tropical_temperate_polar | Coldhouse | Warmhouse | 1 | 3 | 0.12 |  |  |  |
| Northern | collection10_k3_tropical_temperate_polar | Coldhouse | Hothouse | 1 | 0 |  |  |  |  |
| Northern | collection10_k3_tropical_temperate_polar | Coolhouse | Transitional | 1 | 0 |  |  |  |  |
| Northern | collection10_k3_tropical_temperate_polar | Coolhouse | Warmhouse | 1 | 3 | 0.40 |  |  |  |
| Northern | collection10_k3_tropical_temperate_polar | Coolhouse | Hothouse | 1 | 0 |  |  |  |  |
| Northern | collection10_k3_tropical_temperate_polar | Transitional | Warmhouse | 0 | 3 |  |  |  |  |
| Northern | collection10_k3_tropical_temperate_polar | Transitional | Hothouse | 0 | 0 |  |  |  |  |
| Northern | collection10_k3_tropical_temperate_polar | Warmhouse | Hothouse | 3 | 0 |  |  |  |  |
| Southern | occurrence5_k1_tropical_temperate | Coldhouse | Hothouse | 12 | 16 | -0.38 | 40 | 0.005 | 0.050 |
| Southern | occurrence5_k1_tropical_temperate | Transitional | Hothouse | 16 | 16 | -0.16 | 68 | 0.012 | 0.062 |
| Southern | occurrence5_k1_tropical_temperate | Coldhouse | Transitional | 12 | 16 | -0.22 | 57 | 0.037 | 0.092 |
| Southern | occurrence5_k1_tropical_temperate | Coolhouse | Hothouse | 15 | 16 | -0.18 | 73 | 0.033 | 0.092 |
| Southern | occurrence5_k1_tropical_temperate | Coldhouse | Warmhouse | 12 | 13 | -0.29 | 49 | 0.061 | 0.101 |
| Southern | occurrence5_k1_tropical_temperate | Warmhouse | Hothouse | 13 | 16 | -0.08 | 68 | 0.060 | 0.101 |
| Southern | occurrence5_k1_tropical_temperate | Coldhouse | Coolhouse | 12 | 15 | -0.19 | 65 | 0.116 | 0.166 |
| Southern | occurrence5_k1_tropical_temperate | Coolhouse | Transitional | 15 | 16 | -0.03 | 102 | 0.245 | 0.272 |
| Southern | occurrence5_k1_tropical_temperate | Coolhouse | Warmhouse | 15 | 13 | -0.10 | 82 | 0.245 | 0.272 |
| Southern | occurrence5_k1_tropical_temperate | Transitional | Warmhouse | 16 | 13 | -0.07 | 97 | 0.388 | 0.388 |
| Southern | occurrence5_k1_tropical_temperate_polar | Transitional | Hothouse | 12 | 13 | -0.15 | 28 | 0.004 | 0.035 |
| Southern | occurrence5_k1_tropical_temperate_polar | Coolhouse | Hothouse | 11 | 13 | -0.28 | 29 | 0.007 | 0.037 |
| Southern | occurrence5_k1_tropical_temperate_polar | Coldhouse | Hothouse | 5 | 13 | -0.21 | 13 | 0.031 | 0.102 |
| Southern | occurrence5_k1_tropical_temperate_polar | Warmhouse | Hothouse | 11 | 13 | -0.07 | 44 | 0.059 | 0.147 |
| Southern | occurrence5_k1_tropical_temperate_polar | Coolhouse | Warmhouse | 11 | 11 | -0.21 | 40 | 0.095 | 0.189 |
| Southern | occurrence5_k1_tropical_temperate_polar | Coolhouse | Transitional | 11 | 12 | -0.13 | 46 | 0.115 | 0.192 |
| Southern | occurrence5_k1_tropical_temperate_polar | Coldhouse | Transitional | 5 | 12 | -0.06 | 22 | 0.215 | 0.268 |
| Southern | occurrence5_k1_tropical_temperate_polar | Coldhouse | Warmhouse | 5 | 11 | -0.14 | 20 | 0.214 | 0.268 |
| Southern | occurrence5_k1_tropical_temperate_polar | Transitional | Warmhouse | 12 | 11 | -0.08 | 55 | 0.259 | 0.288 |
| Southern | occurrence5_k1_tropical_temperate_polar | Coldhouse | Coolhouse | 5 | 11 | 0.07 | 29 | 0.590 | 0.590 |
| Southern | occurrence5_k2_tropical_temperate | Coldhouse | Hothouse | 9 | 12 | -0.20 | 33 | 0.073 | 0.338 |
| Southern | occurrence5_k2_tropical_temperate | Coolhouse | Hothouse | 14 | 12 | -0.15 | 57 | 0.086 | 0.338 |
| Southern | occurrence5_k2_tropical_temperate | Warmhouse | Hothouse | 8 | 12 | -0.18 | 31 | 0.102 | 0.338 |
| Southern | occurrence5_k2_tropical_temperate | Coldhouse | Transitional | 9 | 7 | -0.06 | 22 | 0.170 | 0.341 |
| Southern | occurrence5_k2_tropical_temperate | Transitional | Hothouse | 7 | 12 | -0.13 | 29 | 0.145 | 0.341 |
| Southern | occurrence5_k2_tropical_temperate | Coldhouse | Coolhouse | 9 | 14 | -0.04 | 53 | 0.275 | 0.411 |
| Southern | occurrence5_k2_tropical_temperate | Coolhouse | Transitional | 14 | 7 | -0.02 | 41 | 0.288 | 0.411 |
| Southern | occurrence5_k2_tropical_temperate | Coldhouse | Warmhouse | 9 | 8 | -0.02 | 32 | 0.368 | 0.460 |
| Southern | occurrence5_k2_tropical_temperate | Coolhouse | Warmhouse | 14 | 8 | 0.03 | 58 | 0.568 | 0.631 |
| Southern | occurrence5_k2_tropical_temperate | Transitional | Warmhouse | 7 | 8 | 0.05 | 35 | 0.807 | 0.807 |
| Southern | occurrence5_k2_tropical_temperate_polar | Coolhouse | Hothouse | 9 | 7 | -0.28 | 7 | 0.006 | 0.055 |
| Southern | occurrence5_k2_tropical_temperate_polar | Coolhouse | Warmhouse | 9 | 5 | -0.32 | 8 | 0.031 | 0.155 |
| Southern | occurrence5_k2_tropical_temperate_polar | Coolhouse | Transitional | 9 | 4 | -0.14 | 8 | 0.071 | 0.195 |
| Southern | occurrence5_k2_tropical_temperate_polar | Transitional | Hothouse | 4 | 7 | -0.14 | 6 | 0.078 | 0.195 |
| Southern | occurrence5_k2_tropical_temperate_polar | Coldhouse | Hothouse | 3 | 7 | -0.15 | 6 | 0.181 | 0.362 |
| Southern | occurrence5_k2_tropical_temperate_polar | Coldhouse | Transitional | 3 | 4 | 0.00 | 4 | 0.298 | 0.497 |
| Southern | occurrence5_k2_tropical_temperate_polar | Coldhouse | Warmhouse | 3 | 5 | -0.18 | 7 | 0.500 | 0.556 |
| Southern | occurrence5_k2_tropical_temperate_polar | Transitional | Warmhouse | 4 | 5 | -0.18 | 9 | 0.451 | 0.556 |
| Southern | occurrence5_k2_tropical_temperate_polar | Warmhouse | Hothouse | 5 | 7 | 0.04 | 16 | 0.435 | 0.556 |
| Southern | occurrence5_k2_tropical_temperate_polar | Coldhouse | Coolhouse | 3 | 9 | 0.13 | 20 | 0.902 | 0.902 |
| Southern | occurrence5_k3_tropical_temperate | Warmhouse | Hothouse | 5 | 8 | -0.27 | 4 | 0.012 | 0.116 |
| Southern | occurrence5_k3_tropical_temperate | Coldhouse | Hothouse | 4 | 8 | -0.23 | 5 | 0.037 | 0.124 |
| Southern | occurrence5_k3_tropical_temperate | Transitional | Hothouse | 4 | 8 | -0.22 | 4 | 0.025 | 0.124 |
| Southern | occurrence5_k3_tropical_temperate | Coldhouse | Coolhouse | 4 | 6 | -0.11 | 9 | 0.297 | 0.594 |
| Southern | occurrence5_k3_tropical_temperate | Coolhouse | Hothouse | 6 | 8 | -0.12 | 18 | 0.239 | 0.594 |
| Southern | occurrence5_k3_tropical_temperate | Coldhouse | Transitional | 4 | 4 | -0.01 | 8 | 0.557 | 0.882 |
| Southern | occurrence5_k3_tropical_temperate | Coldhouse | Warmhouse | 4 | 5 | 0.04 | 13 | 0.804 | 0.882 |
| Southern | occurrence5_k3_tropical_temperate | Coolhouse | Transitional | 6 | 4 | 0.10 | 15 | 0.772 | 0.882 |
| Southern | occurrence5_k3_tropical_temperate | Coolhouse | Warmhouse | 6 | 5 | 0.15 | 21 | 0.882 | 0.882 |
| Southern | occurrence5_k3_tropical_temperate | Transitional | Warmhouse | 4 | 5 | 0.05 | 13 | 0.804 | 0.882 |
| Southern | occurrence5_k3_tropical_temperate_polar | Transitional | Hothouse | 2 | 5 | -0.14 | 0 | 0.041 | 0.244 |
| Southern | occurrence5_k3_tropical_temperate_polar | Coolhouse | Hothouse | 3 | 5 | -0.14 | 3 | 0.117 | 0.350 |
| Southern | occurrence5_k3_tropical_temperate_polar | Coolhouse | Warmhouse | 3 | 2 | -0.10 | 1 | 0.193 | 0.386 |
| Southern | occurrence5_k3_tropical_temperate_polar | Transitional | Warmhouse | 2 | 2 | -0.11 | 1 | 0.349 | 0.419 |
| Southern | occurrence5_k3_tropical_temperate_polar | Warmhouse | Hothouse | 2 | 5 | -0.03 | 3 | 0.281 | 0.419 |
| Southern | occurrence5_k3_tropical_temperate_polar | Coolhouse | Transitional | 3 | 2 | 0.01 | 3 | 0.614 | 0.614 |
| Southern | occurrence5_k3_tropical_temperate_polar | Coldhouse | Coolhouse | 1 | 3 | 0.02 |  |  |  |
| Southern | occurrence5_k3_tropical_temperate_polar | Coldhouse | Transitional | 1 | 2 | 0.02 |  |  |  |
| Southern | occurrence5_k3_tropical_temperate_polar | Coldhouse | Warmhouse | 1 | 2 | -0.08 |  |  |  |
| Southern | occurrence5_k3_tropical_temperate_polar | Coldhouse | Hothouse | 1 | 5 | -0.12 |  |  |  |
| Southern | occurrence10_k1_tropical_temperate | Coldhouse | Hothouse | 12 | 16 | -0.46 | 40 | 0.005 | 0.050 |
| Southern | occurrence10_k1_tropical_temperate | Coolhouse | Hothouse | 15 | 16 | -0.37 | 70 | 0.025 | 0.090 |
| Southern | occurrence10_k1_tropical_temperate | Transitional | Hothouse | 15 | 16 | -0.23 | 71 | 0.028 | 0.090 |
| Southern | occurrence10_k1_tropical_temperate | Warmhouse | Hothouse | 11 | 16 | -0.13 | 51 | 0.036 | 0.090 |
| Southern | occurrence10_k1_tropical_temperate | Coldhouse | Transitional | 12 | 15 | -0.23 | 57 | 0.056 | 0.097 |
| Southern | occurrence10_k1_tropical_temperate | Coldhouse | Warmhouse | 12 | 11 | -0.33 | 40 | 0.058 | 0.097 |
| Southern | occurrence10_k1_tropical_temperate | Coldhouse | Coolhouse | 12 | 15 | -0.09 | 73 | 0.210 | 0.234 |
| Southern | occurrence10_k1_tropical_temperate | Coolhouse | Transitional | 15 | 15 | -0.14 | 90 | 0.181 | 0.234 |
| Southern | occurrence10_k1_tropical_temperate | Coolhouse | Warmhouse | 15 | 11 | -0.24 | 65 | 0.189 | 0.234 |
| Southern | occurrence10_k1_tropical_temperate | Transitional | Warmhouse | 15 | 11 | -0.09 | 82 | 0.500 | 0.500 |
| Southern | occurrence10_k1_tropical_temperate_polar | Coolhouse | Hothouse | 11 | 13 | -0.39 | 27 | 0.005 | 0.044 |
| Southern | occurrence10_k1_tropical_temperate_polar | Transitional | Hothouse | 11 | 13 | -0.26 | 30 | 0.009 | 0.044 |
| Southern | occurrence10_k1_tropical_temperate_polar | Coldhouse | Hothouse | 5 | 13 | -0.30 | 12 | 0.024 | 0.081 |
| Southern | occurrence10_k1_tropical_temperate_polar | Warmhouse | Hothouse | 10 | 13 | -0.12 | 36 | 0.039 | 0.096 |
| Southern | occurrence10_k1_tropical_temperate_polar | Coolhouse | Warmhouse | 11 | 10 | -0.27 | 33 | 0.065 | 0.130 |
| Southern | occurrence10_k1_tropical_temperate_polar | Coolhouse | Transitional | 11 | 11 | -0.14 | 43 | 0.132 | 0.220 |
| Southern | occurrence10_k1_tropical_temperate_polar | Coldhouse | Warmhouse | 5 | 10 | -0.18 | 17 | 0.179 | 0.256 |
| Southern | occurrence10_k1_tropical_temperate_polar | Transitional | Warmhouse | 11 | 10 | -0.13 | 44 | 0.230 | 0.287 |
| Southern | occurrence10_k1_tropical_temperate_polar | Coldhouse | Transitional | 5 | 11 | -0.04 | 24 | 0.367 | 0.408 |
| Southern | occurrence10_k1_tropical_temperate_polar | Coldhouse | Coolhouse | 5 | 11 | 0.09 | 34 | 0.786 | 0.786 |
| Southern | occurrence10_k2_tropical_temperate | Coldhouse | Hothouse | 8 | 12 | -0.27 | 26 | 0.049 | 0.121 |
| Southern | occurrence10_k2_tropical_temperate | Coolhouse | Hothouse | 13 | 12 | -0.37 | 46 | 0.043 | 0.121 |
| Southern | occurrence10_k2_tropical_temperate | Transitional | Hothouse | 6 | 12 | -0.26 | 14 | 0.022 | 0.121 |
| Southern | occurrence10_k2_tropical_temperate | Warmhouse | Hothouse | 8 | 12 | -0.33 | 25 | 0.041 | 0.121 |
| Southern | occurrence10_k2_tropical_temperate | Coldhouse | Coolhouse | 8 | 13 | 0.10 | 56 | 0.628 | 0.628 |
| Southern | occurrence10_k2_tropical_temperate | Coldhouse | Transitional | 8 | 6 | -0.01 | 23 | 0.474 | 0.628 |
| Southern | occurrence10_k2_tropical_temperate | Coldhouse | Warmhouse | 8 | 8 | 0.06 | 32 | 0.521 | 0.628 |
| Southern | occurrence10_k2_tropical_temperate | Coolhouse | Transitional | 13 | 6 | -0.11 | 34 | 0.347 | 0.628 |
| Southern | occurrence10_k2_tropical_temperate | Coolhouse | Warmhouse | 13 | 8 | -0.04 | 52 | 0.514 | 0.628 |
| Southern | occurrence10_k2_tropical_temperate | Transitional | Warmhouse | 6 | 8 | 0.07 | 26 | 0.627 | 0.628 |
| Southern | occurrence10_k2_tropical_temperate_polar | Coolhouse | Hothouse | 7 | 6 | -0.41 | 0 | 0.002 | 0.017 |
| Southern | occurrence10_k2_tropical_temperate_polar | Coldhouse | Hothouse | 3 | 6 | -0.26 | 2 | 0.047 | 0.155 |
| Southern | occurrence10_k2_tropical_temperate_polar | Transitional | Hothouse | 2 | 6 | -0.26 | 0 | 0.033 | 0.155 |
| Southern | occurrence10_k2_tropical_temperate_polar | Coolhouse | Warmhouse | 7 | 5 | -0.34 | 9 | 0.097 | 0.235 |
| Southern | occurrence10_k2_tropical_temperate_polar | Warmhouse | Hothouse | 5 | 6 | -0.08 | 8 | 0.118 | 0.235 |
| Southern | occurrence10_k2_tropical_temperate_polar | Coolhouse | Transitional | 7 | 2 | -0.15 | 4 | 0.232 | 0.387 |
| Southern | occurrence10_k2_tropical_temperate_polar | Coldhouse | Warmhouse | 3 | 5 | -0.19 | 6 | 0.383 | 0.529 |
| Southern | occurrence10_k2_tropical_temperate_polar | Transitional | Warmhouse | 2 | 5 | -0.19 | 4 | 0.423 | 0.529 |
| Southern | occurrence10_k2_tropical_temperate_polar | Coldhouse | Transitional | 3 | 2 | 0.00 | 3 | 0.614 | 0.682 |
| Southern | occurrence10_k2_tropical_temperate_polar | Coldhouse | Coolhouse | 3 | 7 | 0.15 | 17 | 0.945 | 0.945 |
| Southern | occurrence10_k3_tropical_temperate | Transitional | Hothouse | 4 | 5 | -0.34 | 1 | 0.019 | 0.093 |
| Southern | occurrence10_k3_tropical_temperate | Warmhouse | Hothouse | 5 | 5 | -0.50 | 1 | 0.011 | 0.093 |
| Southern | occurrence10_k3_tropical_temperate | Coldhouse | Hothouse | 2 | 5 | -0.32 | 1 | 0.088 | 0.222 |
| Southern | occurrence10_k3_tropical_temperate | Coolhouse | Hothouse | 4 | 5 | -0.34 | 4 | 0.089 | 0.222 |
| Southern | occurrence10_k3_tropical_temperate | Coldhouse | Coolhouse | 2 | 4 | 0.02 | 4 | 0.592 | 0.911 |
| Southern | occurrence10_k3_tropical_temperate | Coldhouse | Transitional | 2 | 4 | 0.02 | 4 | 0.592 | 0.911 |
| Southern | occurrence10_k3_tropical_temperate | Coldhouse | Warmhouse | 2 | 5 | 0.17 | 7 | 0.834 | 0.911 |
| Southern | occurrence10_k3_tropical_temperate | Coolhouse | Transitional | 4 | 4 | 0.00 | 9 | 0.667 | 0.911 |
| Southern | occurrence10_k3_tropical_temperate | Coolhouse | Warmhouse | 4 | 5 | 0.15 | 15 | 0.911 | 0.911 |
| Southern | occurrence10_k3_tropical_temperate | Transitional | Warmhouse | 4 | 5 | 0.16 | 14 | 0.865 | 0.911 |
| Southern | occurrence10_k3_tropical_temperate_polar | Coolhouse | Hothouse | 2 | 3 | -0.32 | 0 | 0.074 | 0.223 |
| Southern | occurrence10_k3_tropical_temperate_polar | Transitional | Hothouse | 2 | 3 | -0.29 | 0 | 0.074 | 0.223 |
| Southern | occurrence10_k3_tropical_temperate_polar | Warmhouse | Hothouse | 2 | 3 | -0.21 | 1 | 0.193 | 0.386 |
| Southern | occurrence10_k3_tropical_temperate_polar | Coolhouse | Warmhouse | 2 | 2 | -0.11 | 1 | 0.349 | 0.524 |
| Southern | occurrence10_k3_tropical_temperate_polar | Coolhouse | Transitional | 2 | 2 | -0.03 | 2 | 0.651 | 0.651 |
| Southern | occurrence10_k3_tropical_temperate_polar | Transitional | Warmhouse | 2 | 2 | -0.08 | 2 | 0.651 | 0.651 |
| Southern | occurrence10_k3_tropical_temperate_polar | Coldhouse | Coolhouse | 0 | 2 |  |  |  |  |
| Southern | occurrence10_k3_tropical_temperate_polar | Coldhouse | Transitional | 0 | 2 |  |  |  |  |
| Southern | occurrence10_k3_tropical_temperate_polar | Coldhouse | Warmhouse | 0 | 2 |  |  |  |  |
| Southern | occurrence10_k3_tropical_temperate_polar | Coldhouse | Hothouse | 0 | 3 |  |  |  |  |
| Southern | collection5_k1_tropical_temperate | Coldhouse | Hothouse | 12 | 13 | -0.47 | 22 | 0.001 | 0.013 |
| Southern | collection5_k1_tropical_temperate | Coolhouse | Hothouse | 15 | 13 | -0.33 | 45 | 0.008 | 0.042 |
| Southern | collection5_k1_tropical_temperate | Coldhouse | Warmhouse | 12 | 11 | -0.34 | 31 | 0.017 | 0.048 |
| Southern | collection5_k1_tropical_temperate | Transitional | Hothouse | 15 | 13 | -0.26 | 52 | 0.019 | 0.048 |
| Southern | collection5_k1_tropical_temperate | Coldhouse | Transitional | 12 | 15 | -0.21 | 59 | 0.068 | 0.098 |
| Southern | collection5_k1_tropical_temperate | Coolhouse | Warmhouse | 15 | 11 | -0.19 | 52 | 0.060 | 0.098 |
| Southern | collection5_k1_tropical_temperate | Warmhouse | Hothouse | 11 | 13 | -0.14 | 45 | 0.066 | 0.098 |
| Southern | collection5_k1_tropical_temperate | Coldhouse | Coolhouse | 12 | 15 | -0.15 | 66 | 0.126 | 0.157 |
| Southern | collection5_k1_tropical_temperate | Coolhouse | Transitional | 15 | 15 | -0.06 | 91 | 0.192 | 0.213 |
| Southern | collection5_k1_tropical_temperate | Transitional | Warmhouse | 15 | 11 | -0.13 | 68 | 0.234 | 0.234 |
| Southern | collection5_k1_tropical_temperate_polar | Coolhouse | Warmhouse | 10 | 8 | -0.42 | 7 | 0.002 | 0.010 |
| Southern | collection5_k1_tropical_temperate_polar | Coolhouse | Hothouse | 10 | 11 | -0.50 | 12 | 0.001 | 0.010 |
| Southern | collection5_k1_tropical_temperate_polar | Transitional | Hothouse | 11 | 11 | -0.30 | 27 | 0.015 | 0.050 |
| Southern | collection5_k1_tropical_temperate_polar | Transitional | Warmhouse | 11 | 8 | -0.22 | 20 | 0.026 | 0.065 |
| Southern | collection5_k1_tropical_temperate_polar | Coldhouse | Hothouse | 5 | 11 | -0.31 | 11 | 0.035 | 0.070 |
| Southern | collection5_k1_tropical_temperate_polar | Coolhouse | Transitional | 10 | 11 | -0.21 | 30 | 0.042 | 0.070 |
| Southern | collection5_k1_tropical_temperate_polar | Coldhouse | Warmhouse | 5 | 8 | -0.23 | 9 | 0.062 | 0.089 |
| Southern | collection5_k1_tropical_temperate_polar | Warmhouse | Hothouse | 8 | 11 | -0.08 | 34 | 0.216 | 0.270 |
| Southern | collection5_k1_tropical_temperate_polar | Coldhouse | Transitional | 5 | 11 | -0.01 | 27 | 0.500 | 0.556 |
| Southern | collection5_k1_tropical_temperate_polar | Coldhouse | Coolhouse | 5 | 10 | 0.20 | 32 | 0.821 | 0.821 |
| Southern | collection5_k2_tropical_temperate | Coolhouse | Hothouse | 11 | 9 | -0.52 | 14 | 0.004 | 0.039 |
| Southern | collection5_k2_tropical_temperate | Coldhouse | Hothouse | 5 | 9 | -0.40 | 6 | 0.016 | 0.082 |
| Southern | collection5_k2_tropical_temperate | Transitional | Hothouse | 6 | 9 | -0.33 | 12 | 0.044 | 0.113 |
| Southern | collection5_k2_tropical_temperate | Warmhouse | Hothouse | 7 | 9 | -0.28 | 15 | 0.045 | 0.113 |
| Southern | collection5_k2_tropical_temperate | Coolhouse | Warmhouse | 11 | 7 | -0.24 | 23 | 0.087 | 0.174 |
| Southern | collection5_k2_tropical_temperate | Coolhouse | Transitional | 11 | 6 | -0.19 | 23 | 0.170 | 0.283 |
| Southern | collection5_k2_tropical_temperate | Coldhouse | Transitional | 5 | 6 | -0.07 | 12 | 0.324 | 0.463 |
| Southern | collection5_k2_tropical_temperate | Coldhouse | Warmhouse | 5 | 7 | -0.12 | 15 | 0.373 | 0.466 |
| Southern | collection5_k2_tropical_temperate | Transitional | Warmhouse | 6 | 7 | -0.05 | 23 | 0.640 | 0.711 |
| Southern | collection5_k2_tropical_temperate | Coldhouse | Coolhouse | 5 | 11 | 0.12 | 32 | 0.714 | 0.714 |
| Southern | collection5_k2_tropical_temperate_polar | Coolhouse | Hothouse | 7 | 4 | -0.40 | 2 | 0.015 | 0.149 |
| Southern | collection5_k2_tropical_temperate_polar | Coldhouse | Hothouse | 3 | 4 | -0.19 | 2 | 0.108 | 0.216 |
| Southern | collection5_k2_tropical_temperate_polar | Coolhouse | Transitional | 7 | 4 | -0.19 | 6 | 0.078 | 0.216 |
| Southern | collection5_k2_tropical_temperate_polar | Coolhouse | Warmhouse | 7 | 4 | -0.32 | 5 | 0.054 | 0.216 |
| Southern | collection5_k2_tropical_temperate_polar | Transitional | Hothouse | 4 | 4 | -0.20 | 3 | 0.097 | 0.216 |
| Southern | collection5_k2_tropical_temperate_polar | Warmhouse | Hothouse | 4 | 4 | -0.08 | 5 | 0.235 | 0.392 |
| Southern | collection5_k2_tropical_temperate_polar | Coldhouse | Warmhouse | 3 | 4 | -0.11 | 5 | 0.430 | 0.614 |
| Southern | collection5_k2_tropical_temperate_polar | Coldhouse | Transitional | 3 | 4 | 0.02 | 6 | 0.570 | 0.634 |
| Southern | collection5_k2_tropical_temperate_polar | Transitional | Warmhouse | 4 | 4 | -0.13 | 8 | 0.557 | 0.634 |
| Southern | collection5_k2_tropical_temperate_polar | Coldhouse | Coolhouse | 3 | 7 | 0.21 | 18 | 0.966 | 0.966 |
| Southern | collection5_k3_tropical_temperate | Coolhouse | Hothouse | 3 | 5 | -0.40 | 0 | 0.018 | 0.110 |
| Southern | collection5_k3_tropical_temperate | Transitional | Hothouse | 3 | 5 | -0.47 | 1 | 0.037 | 0.110 |
| Southern | collection5_k3_tropical_temperate | Transitional | Warmhouse | 3 | 4 | -0.21 | 2 | 0.108 | 0.203 |
| Southern | collection5_k3_tropical_temperate | Warmhouse | Hothouse | 4 | 5 | -0.25 | 5 | 0.135 | 0.203 |
| Southern | collection5_k3_tropical_temperate | Coolhouse | Warmhouse | 3 | 4 | -0.14 | 3 | 0.188 | 0.226 |
| Southern | collection5_k3_tropical_temperate | Coolhouse | Transitional | 3 | 3 | 0.07 | 6 | 0.809 | 0.809 |
| Southern | collection5_k3_tropical_temperate | Coldhouse | Coolhouse | 0 | 3 |  |  |  |  |
| Southern | collection5_k3_tropical_temperate | Coldhouse | Transitional | 0 | 3 |  |  |  |  |
| Southern | collection5_k3_tropical_temperate | Coldhouse | Warmhouse | 0 | 4 |  |  |  |  |
| Southern | collection5_k3_tropical_temperate | Coldhouse | Hothouse | 0 | 5 |  |  |  |  |
| Southern | collection5_k3_tropical_temperate_polar | Coolhouse | Hothouse | 2 | 3 | -0.38 | 0 | 0.074 | 0.223 |
| Southern | collection5_k3_tropical_temperate_polar | Transitional | Hothouse | 2 | 3 | -0.37 | 1 | 0.193 | 0.290 |
| Southern | collection5_k3_tropical_temperate_polar | Coolhouse | Transitional | 2 | 2 | -0.02 | 2 | 0.651 | 0.651 |
| Southern | collection5_k3_tropical_temperate_polar | Coldhouse | Coolhouse | 0 | 2 |  |  |  |  |
| Southern | collection5_k3_tropical_temperate_polar | Coldhouse | Transitional | 0 | 2 |  |  |  |  |
| Southern | collection5_k3_tropical_temperate_polar | Coldhouse | Warmhouse | 0 | 1 |  |  |  |  |
| Southern | collection5_k3_tropical_temperate_polar | Coldhouse | Hothouse | 0 | 3 |  |  |  |  |
| Southern | collection5_k3_tropical_temperate_polar | Coolhouse | Warmhouse | 2 | 1 | -0.26 |  |  |  |
| Southern | collection5_k3_tropical_temperate_polar | Transitional | Warmhouse | 2 | 1 | -0.24 |  |  |  |
| Southern | collection5_k3_tropical_temperate_polar | Warmhouse | Hothouse | 1 | 3 | -0.12 |  |  |  |
| Southern | collection10_k1_tropical_temperate | Coolhouse | Hothouse | 13 | 11 | -0.87 | 14 | 0.000 | 0.005 |
| Southern | collection10_k1_tropical_temperate | Coldhouse | Hothouse | 7 | 11 | -0.53 | 9 | 0.004 | 0.011 |
| Southern | collection10_k1_tropical_temperate | Transitional | Hothouse | 11 | 11 | -0.53 | 20 | 0.004 | 0.011 |
| Southern | collection10_k1_tropical_temperate | Warmhouse | Hothouse | 7 | 11 | -0.47 | 9 | 0.004 | 0.011 |
| Southern | collection10_k1_tropical_temperate | Coolhouse | Warmhouse | 13 | 7 | -0.40 | 22 | 0.034 | 0.068 |
| Southern | collection10_k1_tropical_temperate | Coolhouse | Transitional | 13 | 11 | -0.34 | 52 | 0.135 | 0.226 |
| Southern | collection10_k1_tropical_temperate | Coldhouse | Warmhouse | 7 | 7 | -0.07 | 19 | 0.261 | 0.329 |
| Southern | collection10_k1_tropical_temperate | Transitional | Warmhouse | 11 | 7 | -0.06 | 31 | 0.263 | 0.329 |
| Southern | collection10_k1_tropical_temperate | Coldhouse | Transitional | 7 | 11 | 0.00 | 37 | 0.464 | 0.515 |
| Southern | collection10_k1_tropical_temperate | Coldhouse | Coolhouse | 7 | 13 | 0.34 | 60 | 0.883 | 0.883 |
| Southern | collection10_k1_tropical_temperate_polar | Coolhouse | Hothouse | 7 | 5 | -0.69 | 2 | 0.007 | 0.037 |
| Southern | collection10_k1_tropical_temperate_polar | Transitional | Hothouse | 7 | 5 | -0.46 | 2 | 0.007 | 0.037 |
| Southern | collection10_k1_tropical_temperate_polar | Coolhouse | Warmhouse | 7 | 4 | -0.48 | 4 | 0.036 | 0.121 |
| Southern | collection10_k1_tropical_temperate_polar | Coldhouse | Hothouse | 5 | 5 | -0.17 | 6 | 0.105 | 0.210 |
| Southern | collection10_k1_tropical_temperate_polar | Warmhouse | Hothouse | 4 | 5 | -0.21 | 4 | 0.089 | 0.210 |
| Southern | collection10_k1_tropical_temperate_polar | Coolhouse | Transitional | 7 | 7 | -0.23 | 16 | 0.153 | 0.256 |
| Southern | collection10_k1_tropical_temperate_polar | Transitional | Warmhouse | 7 | 4 | -0.25 | 9 | 0.198 | 0.282 |
| Southern | collection10_k1_tropical_temperate_polar | Coldhouse | Warmhouse | 5 | 4 | 0.04 | 8 | 0.357 | 0.446 |
| Southern | collection10_k1_tropical_temperate_polar | Coldhouse | Transitional | 5 | 7 | 0.29 | 23 | 0.835 | 0.928 |
| Southern | collection10_k1_tropical_temperate_polar | Coldhouse | Coolhouse | 5 | 7 | 0.52 | 26 | 0.928 | 0.928 |
| Southern | collection10_k2_tropical_temperate | Warmhouse | Hothouse | 7 | 5 | -0.89 | 0 | 0.003 | 0.029 |
| Southern | collection10_k2_tropical_temperate | Coolhouse | Hothouse | 5 | 5 | -1.12 | 0 | 0.006 | 0.030 |
| Southern | collection10_k2_tropical_temperate | Coldhouse | Hothouse | 3 | 5 | -0.76 | 0 | 0.018 | 0.061 |
| Southern | collection10_k2_tropical_temperate | Coolhouse | Warmhouse | 5 | 7 | -0.23 | 8 | 0.072 | 0.180 |
| Southern | collection10_k2_tropical_temperate | Transitional | Hothouse | 4 | 5 | -0.97 | 5 | 0.135 | 0.270 |
| Southern | collection10_k2_tropical_temperate | Coolhouse | Transitional | 5 | 4 | -0.15 | 8 | 0.357 | 0.555 |
| Southern | collection10_k2_tropical_temperate | Transitional | Warmhouse | 4 | 7 | -0.08 | 12 | 0.388 | 0.555 |
| Southern | collection10_k2_tropical_temperate | Coldhouse | Coolhouse | 3 | 5 | 0.36 | 15 | 0.991 | 0.991 |
| Southern | collection10_k2_tropical_temperate | Coldhouse | Transitional | 3 | 4 | 0.20 | 9 | 0.892 | 0.991 |
| Southern | collection10_k2_tropical_temperate | Coldhouse | Warmhouse | 3 | 7 | 0.12 | 16 | 0.914 | 0.991 |
| Southern | collection10_k2_tropical_temperate_polar | Coldhouse | Hothouse | 2 | 2 | -0.36 | 0 | 0.123 | 0.245 |
| Southern | collection10_k2_tropical_temperate_polar | Coolhouse | Hothouse | 2 | 2 | -0.63 | 0 | 0.123 | 0.245 |
| Southern | collection10_k2_tropical_temperate_polar | Warmhouse | Hothouse | 4 | 2 | -0.40 | 0 | 0.053 | 0.245 |
| Southern | collection10_k2_tropical_temperate_polar | Coolhouse | Warmhouse | 2 | 4 | -0.24 | 2 | 0.244 | 0.366 |
| Southern | collection10_k2_tropical_temperate_polar | Coldhouse | Warmhouse | 2 | 4 | 0.04 | 4 | 0.592 | 0.710 |
| Southern | collection10_k2_tropical_temperate_polar | Coldhouse | Coolhouse | 2 | 2 | 0.28 | 4 | 0.974 | 0.974 |
| Southern | collection10_k2_tropical_temperate_polar | Coldhouse | Transitional | 2 | 1 | 0.11 |  |  |  |
| Southern | collection10_k2_tropical_temperate_polar | Coolhouse | Transitional | 2 | 1 | -0.17 |  |  |  |
| Southern | collection10_k2_tropical_temperate_polar | Transitional | Warmhouse | 1 | 4 | -0.07 |  |  |  |
| Southern | collection10_k2_tropical_temperate_polar | Transitional | Hothouse | 1 | 2 | -0.47 |  |  |  |
| Southern | collection10_k3_tropical_temperate | Coolhouse | Hothouse | 3 | 3 | -0.75 | 0 | 0.040 | 0.121 |
| Southern | collection10_k3_tropical_temperate | Warmhouse | Hothouse | 4 | 3 | -0.63 | 0 | 0.026 | 0.121 |
| Southern | collection10_k3_tropical_temperate | Transitional | Hothouse | 2 | 3 | -0.75 | 0 | 0.074 | 0.149 |
| Southern | collection10_k3_tropical_temperate | Coolhouse | Warmhouse | 3 | 4 | -0.12 | 4 | 0.298 | 0.358 |
| Southern | collection10_k3_tropical_temperate | Transitional | Warmhouse | 2 | 4 | -0.12 | 2 | 0.244 | 0.358 |
| Southern | collection10_k3_tropical_temperate | Coolhouse | Transitional | 3 | 2 | 0.00 | 3 | 0.614 | 0.614 |
| Southern | collection10_k3_tropical_temperate | Coldhouse | Coolhouse | 0 | 3 |  |  |  |  |
| Southern | collection10_k3_tropical_temperate | Coldhouse | Transitional | 0 | 2 |  |  |  |  |
| Southern | collection10_k3_tropical_temperate | Coldhouse | Warmhouse | 0 | 4 |  |  |  |  |
| Southern | collection10_k3_tropical_temperate | Coldhouse | Hothouse | 0 | 3 |  |  |  |  |
| Southern | collection10_k3_tropical_temperate_polar | Coolhouse | Hothouse | 2 | 2 | -0.63 | 0 | 0.123 | 0.123 |
| Southern | collection10_k3_tropical_temperate_polar | Coldhouse | Coolhouse | 0 | 2 |  |  |  |  |
| Southern | collection10_k3_tropical_temperate_polar | Coldhouse | Transitional | 0 | 1 |  |  |  |  |
| Southern | collection10_k3_tropical_temperate_polar | Coldhouse | Warmhouse | 0 | 1 |  |  |  |  |
| Southern | collection10_k3_tropical_temperate_polar | Coldhouse | Hothouse | 0 | 2 |  |  |  |  |
| Southern | collection10_k3_tropical_temperate_polar | Coolhouse | Transitional | 2 | 1 | -0.17 |  |  |  |
| Southern | collection10_k3_tropical_temperate_polar | Coolhouse | Warmhouse | 2 | 1 | -0.31 |  |  |  |
| Southern | collection10_k3_tropical_temperate_polar | Transitional | Warmhouse | 1 | 1 | -0.15 |  |  |  |
| Southern | collection10_k3_tropical_temperate_polar | Transitional | Hothouse | 1 | 2 | -0.47 |  |  |  |
| Southern | collection10_k3_tropical_temperate_polar | Warmhouse | Hothouse | 1 | 2 | -0.32 |  |  |  |

**Supplementary reference**

1.	Judd EJ, Tierney JE, Lunt DJ, Montañez IP, Huber BT, Wing SL, Valdes PJ. 2024 A 485-million-year history of Earth’s surface temperature. *Science* **385**, eadk3705. (doi:10.1126/science.adk3705)
